# Improving the validity of neuroimaging decoding tests of invariant and configural neural representation

**DOI:** 10.1371/journal.pcbi.1010819

**Published:** 2023-01-23

**Authors:** Fabian A. Soto, Sanjay Narasiwodeyar

**Affiliations:** Department of Psychology, Florida International University, Miami, Florida, United States of America; Kings College London, UNITED KINGDOM

## Abstract

Many research questions in sensory neuroscience involve determining whether the neural representation of a stimulus property is invariant or specific to a particular stimulus context (e.g., Is object representation invariant to translation? Is the representation of a face feature specific to the context of other face features?). Between these two extremes, representations may also be context-tolerant or context-sensitive. Most neuroimaging studies have used operational tests in which a target property is inferred from a significant test against the null hypothesis of the opposite property. For example, the popular cross-classification test concludes that representations are invariant or tolerant when the null hypothesis of specificity is rejected. A recently developed neurocomputational theory suggests two insights regarding such tests. First, tests against the null of context-specificity, and for the alternative of context-invariance, are prone to false positives due to the way in which the underlying neural representations are transformed into indirect measurements in neuroimaging studies. Second, jointly performing tests against the nulls of invariance and specificity allows one to reach more precise and valid conclusions about the underlying representations, particularly when the null of invariance is tested using the fine-grained information from classifier decision variables rather than only accuracies (i.e., using the decoding separability test). Here, we provide empirical and computational evidence supporting both of these theoretical insights. In our empirical study, we use encoding of orientation and spatial position in primary visual cortex as a case study, as previous research has established that these properties are encoded in a context-sensitive way. Using fMRI decoding, we show that the cross-classification test produces false-positive conclusions of invariance, but that more valid conclusions can be reached by jointly performing tests against the null of invariance. The results of two simulations further support both of these conclusions. We conclude that more valid inferences about invariance or specificity of neural representations can be reached by jointly testing against both hypotheses, and using neurocomputational theory to guide the interpretation of results.

## Author summary

Many research questions in sensory neuroscience involve determining whether the representation of a stimulus property is invariant or specific to a change in stimulus context (e.g., translation-invariant object representation; configural representation of face features). Between these two extremes, representations may also be context-tolerant or context-sensitive. Most neuroimaging research has studied invariance using operational tests, among which the most widely used in recent years is cross-classification. We provide evidence from a functional MRI study, simulations, and theoretical results supporting two insights regarding such tests: (1) tests that seek to provide evidence for invariance (like cross-classification) have an inflated false positive rate, but (2) using complementary tests that seek evidence for context-specificity leads to more valid conclusions.

## Introduction

**Fig 1:**
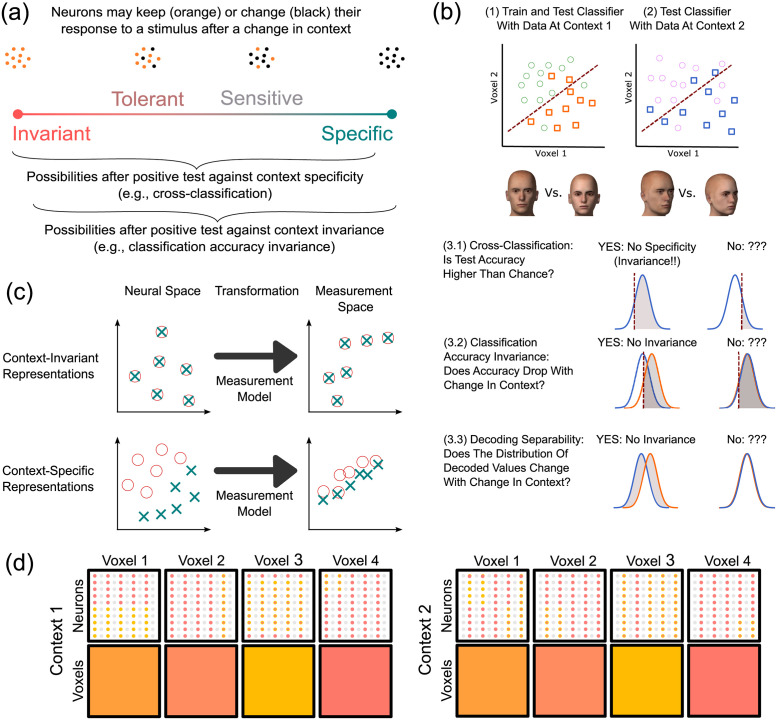
Tests of invariant and configural brain representation. A: Varying degrees of change in neural encoding as a function of a change in context. With a change in context, context-invariant representations do not change at all, whereas context-specific representations change completely, with a continuum between both extremes. Tests in the literature focus on evidence against one of the two extremes. B: Tests of context invariance and specificity. Steps 1 and 2 are common to all tests. Different tests differ on how invariance/specificity is evaluated in step 3. The figure depicts distributions of classifier decision variables and the areas of these distributions on which each test focuses (in gray). C: Representations are transformed from the space of neural activities to the space of voxel measurements. Context-invariant representations (top) cannot be transformed to decrease their invariance and increase their specificity, whereas context-specific representations (bottom) can be transformed to increase their invariance and decrease their specificity. D: Example highlighting the differences between spatially smooth versus fine-grained encoding schemes, and a particular combination of the two schemes that produces false-positives in a voxelwise analysis. Each column represents a voxel containing neurons (small circles), each with selectivity for one of two values of the target property (red and yellow). The multivoxel pattern of activity is the same for both levels of the context dimension (spatially smooth encoding), but completely different populations of neurons encode each level (fine-grained encoding). This figure includes public domain clipart and all other parts are original: https://commons.wikimedia.org/wiki/File:Gaussian_distribution.svg https://www.wpclipart.com/signs_symbol/arrows/BW_arrows/arrow_BW_thick_left.png.html.

A common question in sensory and cognitive neuroscience is to what extent the neural representation of a stimulus property changes as a function of changes in other aspects of stimulation–that is, the context in which it is presented. As shown in Fig 1A, one possibility is that the neural representation of the target property is invariant to changes in context. In that case, the neural activity representing the target property does not change at all with changes in context. Another possibility is that the neural representation of the target property is context-specific. In that case, the neural activity representing the target property completely changes with a change in context. Another way to describe context-specificity is by saying that the target property and its context are represented configurally; that is, as a configuration separate from its components. As shown in Fig 1A, these two cases of complete invariance and specificity should be seen as extremes in a continuum. In this continuum, representations that are closer to invariance (left half of the continuum) could be characterized as “tolerant” to changes in context, whereas representations that are closer to specificity (right half of the continuum) could be characterized as “sensitive” to changes in context.

Most human neuroimaging research has studied invariance and specificity using operational tests that provide evidence against the null hypotheses represented by the two extremes in Fig 1A. However, most neuroscientists are more interested in determining to what extent a representation is closer to one of the extremes in the continuum, being classified as either context-tolerant or context-sensitive.

For example, probably the most widely used test in this area is cross-classification (or cross-decoding; [1–3]; we have also called this test classification accuracy generalization: [4]), illustrated in Fig 1B. The first step in cross-classification is to train a classifier to decode a particular stimulus feature, such as whether a presented face is male or female, from patterns of fMRI activity observed across voxels. The second step is to test the trained classifier with new patterns of fMRI activity, this time obtained from presentation of the same stimuli, but changed in an irrelevant property, such as head orientation. Using our nomenclature, in this example the target stimulus property is face sex, and the context is face orientation. If accuracy with the test data is higher than chance, then researchers usually conclude that the neural representation of the target feature has a certain level of tolerance to changes in context (usually described as invariance), within the area from which the fMRI activity was obtained.

The cross-classification test has been used to provide evidence for tolerant encoding of face identity across viewpoint [5], object category and viewpoint across spatial position [6], object category across shape (and vice-versa; [7]), motor actions across modalities [8], place of speech articulation features across manner of articulation [9], object category [10] or face identity [11] across stimulus modality, word semantic category across stimulus modality [12], learned category labels across categorization tasks [13], and semantic word representation across languages [14], among others (for a review, see [3]).

Cross classification is a test against the null hypothesis of no generalization of decoding accuracy from one context to another, a condition that would be met under context-specific encoding of the target property. As shown in Fig 1, evidence against the extreme of context-specificity means that the representation can fall anywhere in the continuum except the right extreme. Invariance and tolerance are only some of the possibilities, as representations may also be context-sensitive.

An example of a test that provides evidence against the null hypothesis of invariance is the *classification accuracy invariance* test [4]. As shown in Fig 1B, this test involves the same steps described for cross-classification, but during the test phase the classifier is presented with data obtained at both the training and the testing contexts (i.e., context 1 and 2 in Fig 1). The null hypothesis is that decoding accuracy is equivalent across contexts (see step 3.2 in Fig 1B). When accuracy drops significantly from training to testing context, one can conclude that the underlying representation of the decoded property is not invariant to context. We are aware of at least one prior study using a version of this test to study encoding of face information [6].

Again, evidence against the extreme of context-invariance means that the representation can fall anywhere else in the continuum shown in Fig 1A. Context specificity is only one of the possibilities, as representations may also be context-sensitive or context-tolerant.

An important issue in current practice is that researchers seem to believe that invariance and specificity can be contrasted with each other, ignoring that a continuum exists between those two extremes and most cases are likely to lie somewhere in that continuum. Thus, a more reasonable approach would be to determine whether enough evidence exists to reject one extreme and not the other, which provides evidence that the representation lies either at the left half of the continuum (invariance/tolerance) or at the right half (specificity/sensitivity).

In a previous theoretical paper [4], we explored to what extent the context tolerance or specificity of neural representations could be measured using a variety of neuroimaging analyses, with a focus on decoding tests like cross-classification and classification accuracy invariance. Because neuroimaging involves only indirect measures of neural activity, it cannot be used to get precise indicators of where a neural representation falls within the continuum shown in Fig 1A. In general, the process by which neural representations are transformed from the neural space into a space of measurements (e.g., voxel activities) will distort the representations in such a way that makes such precise indicators impossible. However, the results of neuroimaging decoding tests like those just described do allow to make some inferences about the underlying neural representations. Besides clarifying what different tests measure (i.e., cross-classification provides evidence *against* context-specificity, rather than evidence *for* invariance), this theoretical work provides two important insights that have consequences for neuroimaging research.

The first theoretical insight, which was not explicitly described or supported in our previous work but is strongly suggested by that work, is that jointly performing tests against the nulls of invariance and specificity allows one to reach more precise and valid conclusions about the underlying representations. When both types of tests are carried out, one can use Table 1 to reach valid conclusions about properties of the underlying neural code. For example, one may use the cross-classification test to obtain evidence against context-specificity, but usually researchers who use this test are interested in reaching a conclusion favoring invariance or tolerance (e.g., [3, 5]). For that, information from a test against invariance would be very useful. If a test against invariance is not significant, one can make a stronger case for tolerant representations. Because sample size and measurement noise are equivalent in this test and the significant cross-classification test, the best interpretation is that the underlying representation is likely to be farther away from specificity than from invariance, being tolerant/invariant rather than sensitive. On the other hand, if the test offers evidence against invariance, then the underlying representations could be anywhere in the continuum shown in Fig 1A, except at the two extremes, and it would be premature to make a conclusion of tolerance in the underlying representations, as they may also be context-sensitive. Because tests against invariance have been rarely used in the literature, one goal of the current study is to provide evidence of the validity of such tests, and for our claim that performing them together with tests against specificity should lead to more valid conclusions about the underlying representations.

**Table 1:** Lookup table summarizing how joint tests against specificity and invariance should be interpreted.

		Test against specificity (e.g., cross-classification)	
		Not significant	Significant
Test against invariance (e.g., decoding separability)	Not significant	Inconclusive results	Tolerance or invariance likely
	Significant	Specificity or sensitivity likely	Inconclusive results

Note that significance of the popular cross-classification test does not guarantee a conclusion for tolerance or invariance. Only when such a test is accompanied by a nonsignificant test against invariance one can reach a positive conclusion.

The second theoretical insight is that there is an important asymmetry regarding the validity of tests of invariance and context-specificity. If the underlying neural representation is truly invariant, then a signal showing evidence against invariance will never be found from neuroimaging decoding tests. In this case, any finding of lack of invariance would result from measurement noise, and the probability of such finding would be equal to the false positive (type I) error rate of the statistical test, usually *α* = .05. On the other hand, if the underlying representation is truly context-specific, it is still possible to find a signal at the level of voxels showing evidence against context-sensitivity. In this case, such a signal will add to the probability of false positives, which would be higher than *α*.

The reason lies in the contribution of the measurement model, which summarizes how representations are transformed from the space of neural representations into the space of measured variables. Fig 1C depicts a schematic example, where representations of the target stimuli in one context (e.g., faces with front orientation) are shown as red circles, and representations in a second context (e.g., faces with sideways orientation) are shown as green crosses. In the top example, the original neural representations are fully context-invariant, meaning that the representation of a stimulus in either context is in the exact same point in neural space. Regardless of what transformation is induced by the measurement model, such representations will remain invariant in the measurement space, as the transformation will have the same effect on two identical representations (i.e., overlapping crosses and circles in Fig 1C). In the bottom example, the original representations are fully context-specific, meaning that the stimulus representations occupy completely different regions of space depending on context. In this case, there are transformations that would reduce differences in the representation of stimuli across contexts, making the representations less context-specific. In sum, the transformation from neural space to measurement space (i.e., the measurement model) cannot make a completely invariant representation appear as if it was sensitive to context, but it can make a completely context-specific representation appear as if it was tolerant to changes in context.

In our previous work [4], we showed through mathematical proofs that this asymmetry is inherent to inferences about invariance and specificity from indirect measures of neural activity. While those results are general (i.e., they make no assumptions about the specifics of encoding and measurement), they are also very abstract and do not allow one to precisely characterize the potential pervasiveness of the problem in neuroimaging studies. For that, one must be more explicit about the specific encoding and measurement models that are assumed to be at play. Here we take a step in this direction by focusing on encoding and measurement models widely used in computational cognitive neuroscience and thought to be at play in neuroimaging studies of encoding in early vision.

The simplest example is one in which changes in the target property produce smooth changes in the spatial distribution of activity, in a similar scale as voxel size, while changes in context produce changes in the fine-grained spatial distribution of activity, at the sub-voxel level [15]. Take the example shown in Fig 1D. Each column represents a different voxel containing a large number of neurons, represented by small circles, with selectivity for some target stimulus property. In this simplified example, the neurons can show preference for one of two values of the target property, represented by the colors red and yellow. Neurons can be inactive in a particular context, which is represented by the color gray. Different voxels have different proportions of the two types of neurons, so that despite of the spatial pooling of activity produced at each voxel, there is a distinctive pattern of activity produced across voxels by each stimulus property. This is a spatially smooth coding scheme.

On the other hand, note how within a voxel widely different spatial distributions of activity may produce the same value of global activity at the voxel level. For example, the same aggregate activity is obtained for voxel 1 in context 1 (top) and context 2 (bottom), despite the fact that the fine-grained distribution of activities is widely different. The same is true for all other voxels. Thus, within each voxel one can see a fine-grained coding scheme that distinguishes between contexts.

More importantly, in Fig 1D the neurons encoding the target dimension in the first context (uneven columns of neurons) are completely different to those encoding the target dimension in the second context (even columns of neurons). However, the spatial distribution of neurons specific to each value of the context dimension is spatially homogeneous, with about the same number of neurons of each kind in the voxel regardless of context.

The result of a spatially smooth encoding of the target dimension across voxels, together with a fine-grained spatial distribution of neurons specific to each value of the context dimension, produce as a result a case in which neural encoding of the target dimension is context-specific, but appears as perfectly invariant at the level of voxel activities.

A good example of this type of encoding in the brain is encoding of spatial position and orientation in V1. Encoding of spatial position is spatially smooth in V1, with the scale of retinotopic maps being similar to the voxel sizes typically used in neuroimaging, whereas encoding of orientation is much more spatially fine-grained (see [16, 17]). This example shows that the kind of encoding scheme exemplified by Fig 1D can be found in the brain.

Because of the influence of the measurement model depicted in Fig 1C, the need to jointly perform and interpret tests of invariance and specificity is even greater for researchers who aim to find evidence for tolerant/invariant representations. If a false positive is found in a test of context-specificity (e.g., cross-classification) due to issues in the measurement model, it is unlikely that a test of invariance (e.g., classification accuracy invariance) will also be significant. The inherent tendency toward false positives (i.e., > *α*) of the cross-classification test can be partially controlled by interpreting its results together with results of tests against the null of invariance.

Here, we show that the two theoretical insights described above have important consequences for neuroimaging research, through empirical evidence coming from an fMRI decoding study, and computational evidence coming from simulation work. In the empirical study, we perform decoding of orientation and spatial position from fMRI activity patterns recorded in V1, a case in which properties of the underlying neural code are known. The cross-classification test provides strong evidence for the incorrect conclusion that, in V1, encoding of spatial position is tolerant/invariant to changes in orientation, as well as some evidence for the incorrect conclusion that orientation is tolerant/invariant to changes in spatial position. We find that the use of theoretically-derived tests of invariance can lead to more valid conclusions regarding the underlying code. The results of two simulations further support all of these conclusions. Our results highlight the validity and value of using tests of invariance together with tests of context-specificity (e.g., cross-classification) when attempting to draw inferences about neural representations from neuroimaging decoding studies.

## Results

### Experimental results

The goal of our study was to validate the two insights provided by neurocomputational theory [4] described above. For this, we applied decoding tests of invariance and specificity to the study of orientation and spatial position in V1. Previous research has established that these properties are not encoded in an invariant way but, as explained earlier, the spatial scale of orientation and spatial position maps in V1 is likely to lead to the incorrect conclusion of invariance if tests of specificity, such as cross-classification, are applied on their own.

**Fig 2:**
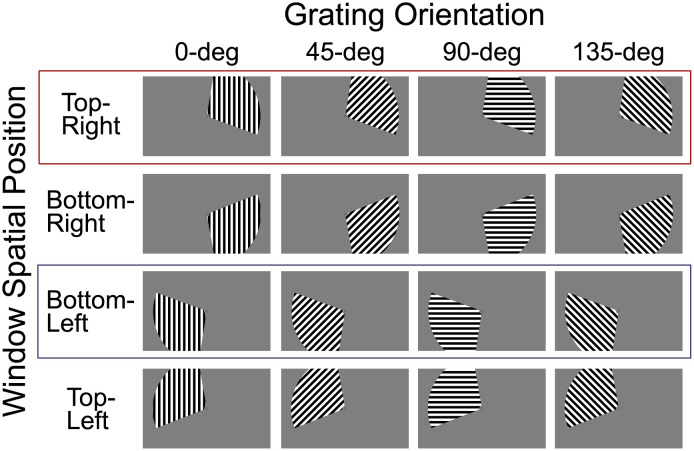
Stimuli. Stimuli were composed of oriented gratings (dimension 1) presented in a windowed spatial position (dimension 2). Each trial consisted of a single combination of oriented gratings and spatial position. Training runs were composed of stimuli presented only in top-right and bottom-left spatial positions (highlighted through red and blue boxes). Testing runs included all sixteen stimulus combinations.

Participants were presented with the stimuli in Fig 2 while they performed a task involving a stimulus presented at the center of the screen. Functional MRI data was acquired at the same time, with separate runs providing data for training and testing of a support vector machine (SM) classifier. Training runs were composed of stimuli presented only in spatial positions top-right and bottom-left (highlighted through red and blue boxes in Fig 2). Testing runs included all sixteen stimulus combinations. We trained a linear SVM classifier to decode a target dimension (e.g., spatial position) while holding the context dimension (e.g., grating orientation) constant. We then tested the classifier with data obtained at the trained value of the context dimension (e.g., 0° orientation) as well as new values of the context dimension (e.g., 45°, 90°, and 135° orientation). The classifier provided decision variables and accuracy estimates used to perform a test of specificity (cross-classification) and two tests of invariance (classification accuracy invariance, decoding separability) presented below (for more details, see Materials and methods).

#### The cross-classification test produces false positives

We performed a set of analyses using the cross-classification test to validate our theoretical prediction that this method should produce findings of false-positive invariance. The cross-classification test was conducted by assessing whether a linear decoder trained to classify the target dimension at one level of the context dimension, could perform the same classification above chance across non-trained levels of the context dimension. A positive result in the cross-classification test is usually taken as evidence for the existence of invariant representations in the area of interest [2, 3].

We conducted two separate analyses using the cross-classification test in which we switched the identities of the target and context dimensions. In the first analysis, spatial position was treated as the target dimension to be decoded, while orientation remained as the context dimension. To obtain decoded stimulus values for spatial position, we used deconvolved single-trial estimates of activity in V1 voxels as input to the SVM linear decoder. We trained the decoder to classify trials based on spatial position labels (top-right vs bottom-left, see boxed stimuli in Fig 2) and holding constant the level of grating orientation (context dimension; for example, 0°) using leave-one-run-out cross-validation, and tested it with independent data sets across all levels of grating orientation (0°, 45°, 90°, and 135°). To test for cross-classification invariance, we performed a binomial test on the accuracy estimates from the testing data set, corrected for multiple comparisons using the Holm-Sidak method (for more details, see *fMRI decoding tests*). If the accuracy score was significantly above chance, then the cross-classification test concludes that spatial position is encoded invariantly from orientation in V1, a conclusion known to be false.

For each participant, we repeated the analysis four times, once for each level of grating orientation that was held fixed in the classifier’s training data. We predicted that the cross-classification test would generate consistent false positives in the case where spatial position was used as the relevant dimension to be decoded. Since spatial position is encoded in a spatially smooth manner in V1, we expected strong performance of the classifier across all levels of orientation. In other words, we expected the accuracy scores of the classifier to remain above chance across different levels of the context dimension.

**Fig 3:**
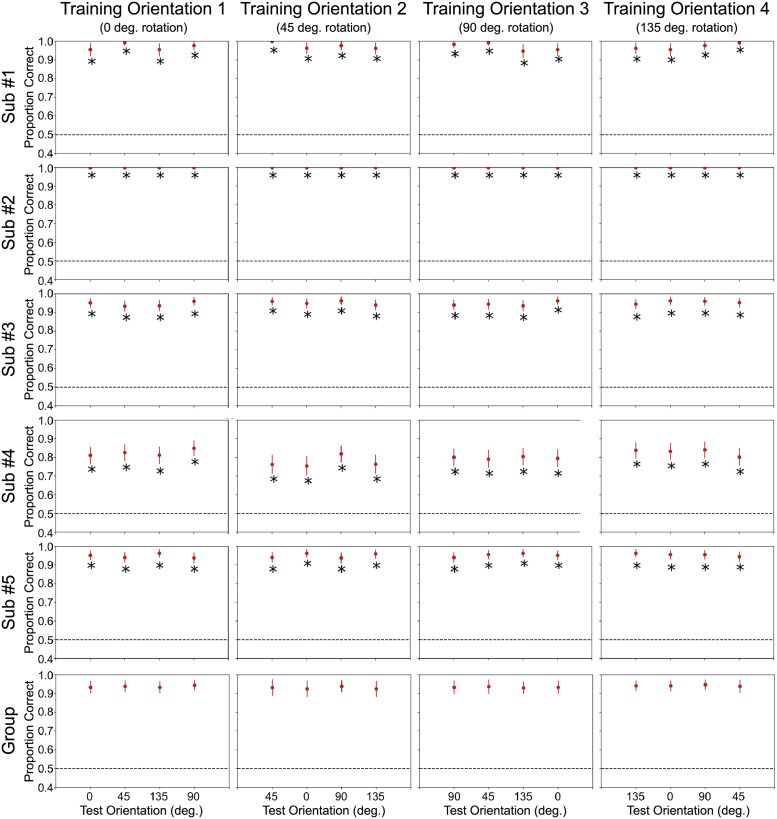
Classification accuracy results with test data for the decoding of spatial position. Each row represents a complete analysis for a single subject. Columns represent different levels of the context dimension (orientation) held fixed during training and the dotted line represents chance performance. Group descriptive statistics (mean and standard errors) are presented in the bottom row. Also shown are results of significant cross-classification and classification accuracy invariance (pairwise comparisons, none significant) tests.

Fig 3 shows accuracy estimates from such a decoding procedure for all five subjects. The SVM linear decoder achieves extremely high levels of classification accuracy in test sets across all 5 subjects. As predicted, the test incorrectly finds evidence for invariance of spatial position from orientation in all participants and all tests (all *p* < .001; for details see Table A in S1 Text). This result is unsurprising, in the sense that one would intuitively expect it given the properties of encoding in V1. The important point, however, is that in most applications of the cross-classification test researchers do not know much about encoding in the area under study, and they could easily conclude in favor of invariance when the underlying code does not show such property.

**Fig 4:**
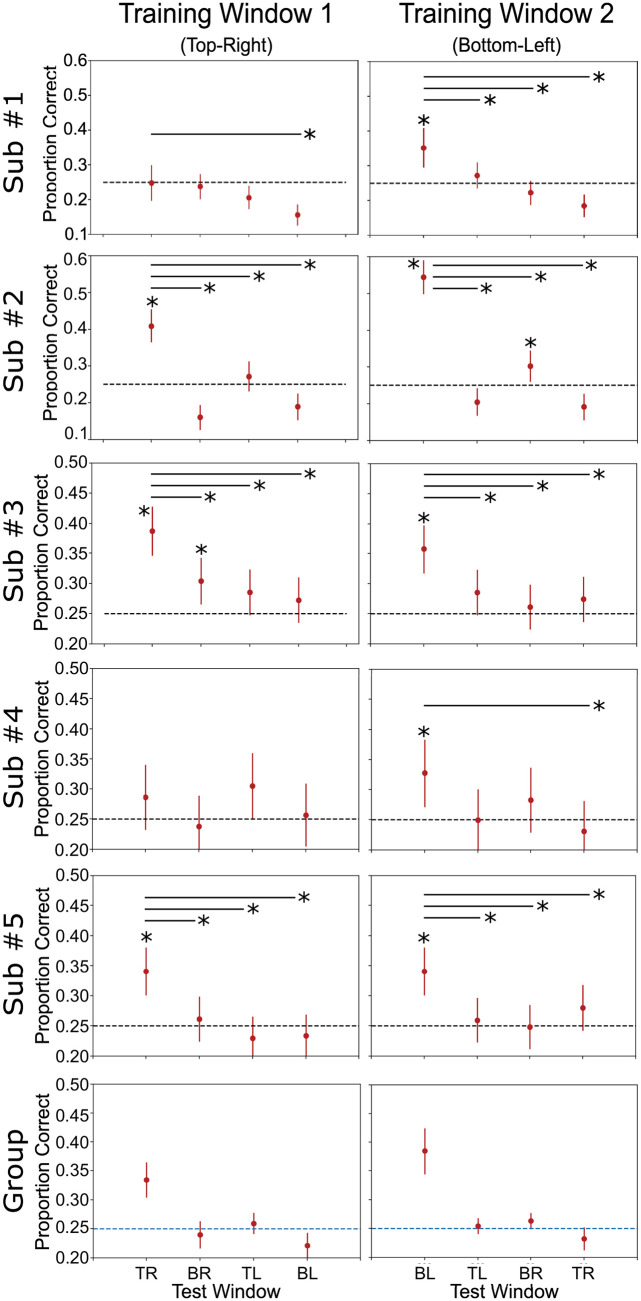
Classification accuracy results with test data for the decoding of orientation. Each row represents a complete analysis for a single subject. Columns represent different levels of the context dimension (spatial position) held fixed during training and the dotted line represents chance performance. Group descriptive statistics (mean and standard errors) are presented in the bottom row. Also shown are results of significant cross-classification and classification accuracy invariance (pairwise comparisons) tests.

We performed a second analysis in which orientation was the target dimension to be decoded, while spatial position was the context dimension. We trained the decoder to classify trials based on grating orientation (0°, 45°, 90°, and 135°, see boxed stimuli in Fig 2) and holding constant the position of the spatial window (context dimension; for example, top-right in Fig 2) using leave-one-run-out cross-validation, and tested it with independent data sets across all levels of spatial position (top-right, bottom-right, bottom-left, and top-left in Fig 2). All other procedures remained the same as in the first analysis. Fig 4 shows decoding accuracy results for the orientation analysis. The SVM classifier was able to successfully decode orientation information at the original training position in all subjects, but for subjects 1 and 4 this was restricted to a single training window (bottom-left), which is at least partially due to individual differences in the quality of data (note that decoding accuracies are lowest for subject 4 in Fig 3). In contrast to spatial position classification, the classifier’s accuracy scores drop significantly in untrained testing windows.

The classifier accuracy at the training window provides a ceiling of performance for the cross-classification accuracy (see [2, 3]). That is, we are not interested in the analyses with non-significant accuracies at the training window (sub#1 and sub#4 at training window 1; see Fig 4), as in those cases we would not expect a significant cross-classification accuracy. Out of the eight analyses showing significant accuracy at the training window, two generated significant cross-classification results, which would lead to an invalid conclusion of invariance. This number was higher than the 5% expected false positive rate for these tests, but a binomial test did not reach significance with *p* = .051, probably due to the low power of a test involving only eight analyses.

#### Jointly testing against specificity and invariance leads to valid conclusions

The results from the previous section showed that the cross-classification test, which tests against the null hypothesis of context-specificity, can lead to erroneous conclusions about invariance of representations. We next aimed to show that the addition of tests of invariance [4] could solve such issues and lead to more valid conclusions about the underlying code. Here, we apply two of these tests on our data set: the classification accuracy invariance test and the decoding separability test. In contrast to the cross-classification test, both of these theoretically-driven tests try to detect failures of invariance as opposed to providing evidence for invariance.

The classification accuracy invariance test defines invariance as the case where the probability of correct classification is exactly the same across all contexts. With invariance being the null hypothesis, the test is sensitive to any drop in the classifier’s performance across different levels of the context dimension. We implemented the classification accuracy invariance test by applying an omnibus Chi-Square test on the accuracy estimates from the linear decoder (i.e., testing whether all proportions are the same or some of them are different). Then, we performed pairwise comparisons between accuracy at the training level and each non-training level of the context dimension.

The decoding separability test, unlike the previous two tests, does not make use of classification accuracy estimates. Instead, it directly relies on certain properties of the decoding probability distributions for individual stimuli. That is, linear classifiers like the one used here perform classification of a new data point by computing a decision variable *z*, representing the distance of the data point from the classifier’s hyperplane separating two classes. When the decision variable is larger than some criterion value (usually zero), the output is one class, whereas when the decision variable is smaller than the criterion the output is the other class. Instead of comparing simple accuracy estimates, the decoding separability test compares the full distributions of such decision variables, or *decoding distributions*.

This test followed the same steps and rationale as the classification accuracy invariance test presented earlier, but instead of computing accuracies and testing their differences, we obtained decision variables from the trained classifier, and used those to estimate decoding distributions using kernel density estimation (see Fig 1B). For each pair of stimuli differing in the context dimension (e.g., 0° and 45° grating orientation, when the decoded variable was spatial position) we computed the distance between decoding distributions using a discretized *L*1 metric, which corresponds to the shadowed area in step 3.3 of Fig 1B. Then, we summed a number of such *L*1 metrics across values of the decoded dimension (e.g., the two spatial windows, when the decoded variable was spatial position), which produced an $L1_{j}^{G}$ statistic (see Eq 3). Simply put, while a single *L*1 metric is analogous to the accuracy of the classifier for a single decoded label, the $L1_{j}^{G}$ statistic is analogous to the overall decoding accuracy across all labels. The only difference is that $L1_{j}^{G}$ measures distances between decoding distributions, rather than accuracies. We performed a permutation test to determine whether the observed $L1_{j}^{G}$ statistic was higher than expected by chance under the null hypothesis of invariance; a positive result on this test gives evidence against neural invariance for the given comparison. Also, we must note that, in theory, the decoding separability test should provide more information about (and be more sensitive to) such violations than the decoding accuracy invariance test (see [4]).

**Fig 5:**
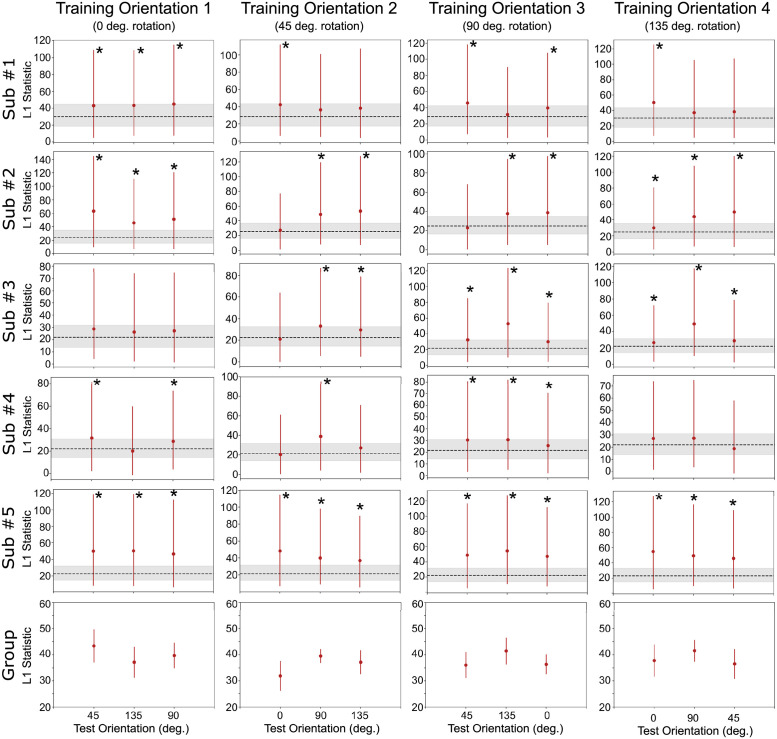
Decoding separability test results with spatial position as the target dimension. Each row represents a complete analysis for a single subject. Columns represent different levels of the context dimension (orientation) during training. The *y*-axis shows the $L1_{j}^{G}$ statistic and bars represent 90% bootstrap confidence intervals. The dotted line and surrounded gray area represent the expected value and 90% bootstrap confidence interval for the $L1_{j}^{G}$ statistic when no differences exist between two distributions. Group descriptive statistics (mean and standard errors) are presented in the bottom row.

As before, we first applied the invariance tests to decoding results from the spatial position classification. Results from the classification accuracy invariance are shown in Fig 3, and results from the decoding separability test are shown in Fig 5. The specific values obtained from the two tests are reported in Tables B and C in S1 Text. The classification accuracy invariance test (Fig 3) did not find evidence against invariance in any of the subjects. However, in line with theoretical predictions, the decoding separability test (Fig 3) was much more sensitive to evidence against invariance present in the data. The test found failures of invariance in many cases where accuracy-based tests either found false positives (i.e., cross-classification) or failed to detect failures of invariance (i.e., classification accuracy invariance; see Fig 3). Overall, we found that the decoding separability test detected failures of invariance in the data of all five participants (17 out of 20 analyses).

From these results, it is apparent that the decoding separability test is sensitive to failures of invariance known to exist in the underlying neural code, even when decoding accuracy seems to suggest perfect invariance (see Fig 3). These results serve as an empirical validation of the decoding separability test, which was developed directly from theory [4]. In addition, these results show the value of testing against invariance, in addition to testing against specificity, to reach valid conclusions about the invariance or specificity of underlying neural representations. Performing both tests and following the guidelines in Table 1, results are inconclusive about whether encoding of spatial position in V1 is invariant or specific to orientation. This conservative conclusion is far better than the invalid conclusion that one would reach by performing the cross-classification test by itself; namely, that encoding of spatial position in V1 is invariant to orientation.

**Fig 6:**
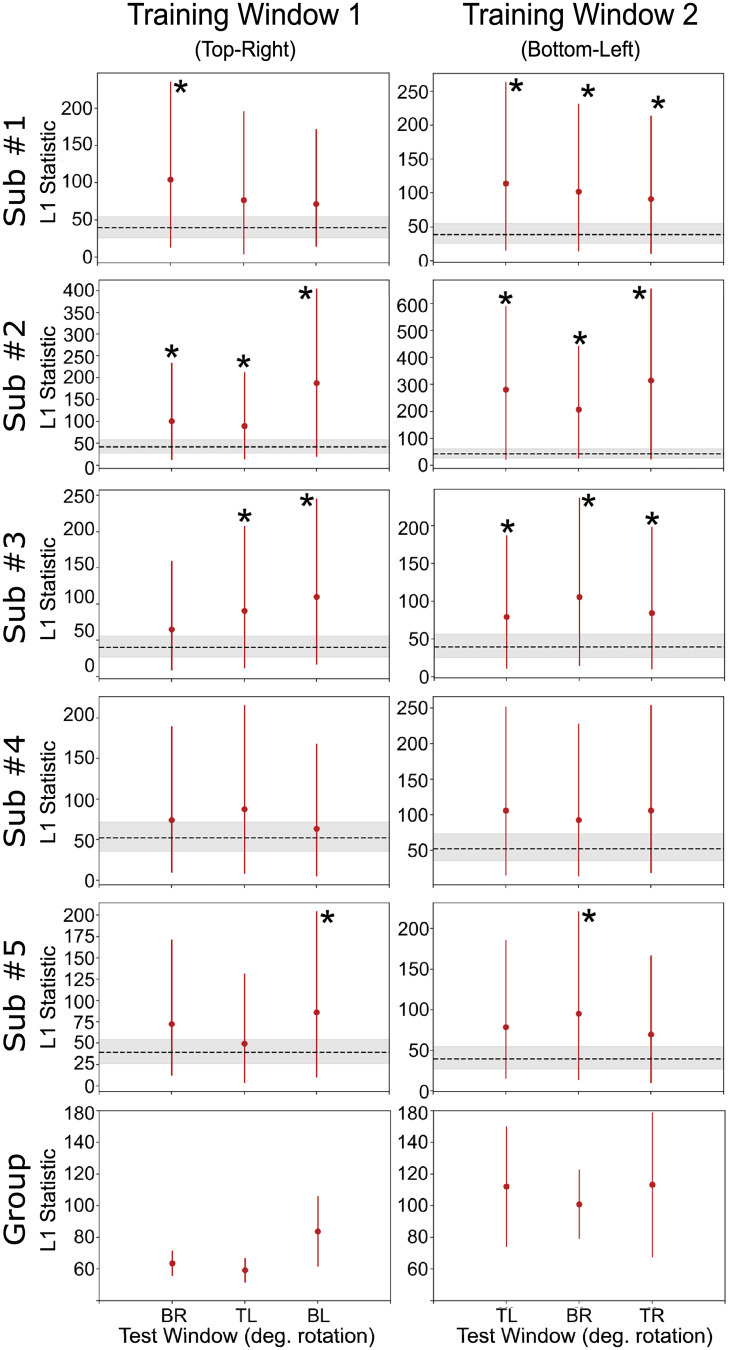
Decoding separability test results with orientation as the target dimension. Each row represents a complete analysis for a single subject. Columns represent different levels of the context dimension (spatial position) during training. Values in the *x*-axis represent levels of the context dimension (spatial position) during testing (TR: top-right; BR: bottom-right; TL: top-left; BL: bottom-left). The *y*-axis shows the $L1_{j}^{G}$ statistic and bars represent 90% bootstrap confidence intervals. The dotted line and surrounded gray area represent the expected value and 90% bootstrap confidence interval for the $L1_{j}^{G}$ statistic when no differences exist between two distributions. Group descriptive statistics (mean and standard errors) are presented in the bottom row.

Next, we applied the invariance test to decoding results from the orientation classification. Results from the classification accuracy invariance test are shown in Fig 4, and results from the decoding separability test are shown in Fig 6. The specific values obtained from the two tests are reported in Tables B and C in S1 Text. The classification accuracy invariance test (Fig 4) was much more sensitive to failures of invariance in this analysis. Failures of invariance were detected in every case where the classifier successfully decoded orientations above chance levels in the training window. Interestingly, failures of invariance were also detected in cases where the classifier did not successfully decode orientation above chance. This is counterintuitive, but expected from a theoretical point of view (see [4]), which suggests that a decoder does not have to perform accurately or be optimal in any way to be able to detect failures of invariance. Contrary to our expectations, in this analysis the decoding separability test detected failures of invariance less frequently than the classification accuracy invariance test (see Fig 6). The decoding separability test detected failures of invariance in the data of four out of five participants (eight out of ten analyses).

In comparison to the classification accuracy invariance test, the decoding separability test appears to be more sensitive to detecting failures of invariance in cases where the decoder’s performance reaches ceiling levels (Fig 5). However, when classification accuracy is well below ceiling levels, as in decoding of orientation, the test seems less sensitive than classification accuracy invariance, perhaps due to a lower statistical power of the permutation test involved.

As was the case with decoding of spatial position, in this second analysis we also see the value of testing against invariance. While the results of the cross-classification test suggested invariant representations in subjects #2 and #3 (see Fig 4), such results are inconclusive when interpreted in the context of tests of invariance.

### Simulation and theoretical results

The empirical results described in the preceding section clearly support the hypotheses that tests aimed at providing evidence for invariance, such as cross-classification, are prone to false positives, and that jointly performing tests against the nulls of invariance and specificity allows one to reach more precise and valid conclusions about the underlying representations.

However, there are issues with experimental work that motivated us to further evaluate our hypotheses through simulation work. In particular, experimental work does not allow full control of the underlying neural representations. In our study, we assumed that encoding of spatial position was specific to orientation, and vice-versa, but it is unlikely that the true encoding of these variables in V1 is completely context-specific. For example, there is evidence that a minority of neurons in V1 are invariant to orientation [18]. This means that encoding of spatial position is best characterized as context-sensitive, but a critical reader could interpret this as evidence for tolerance. Simulation work provides complete control over the representations under study, which can be made to be fully context-specific, without any degree of tolerance to changes in context. The relevant question is: Does cross-classification lead to conclusions of false-positive invariance under such circumstances? If yes: Can tests against invariance lead to more valid conclusions?

Another issue with experimental results is that they can be difficult to generalize. A critical reader could argue that issues with tests of context-specificity like cross-classification are restricted to special cases, and not general as suggested by theory. Again, simulation and theoretical work allows one to provide results that are more general.

#### Simulation 1: False positive invariance resulting from features of the measurement model

Some researchers might argue that they use the cross-classification test to detect *any* level of context-tolerance or context-sensitivity. Indeed, some researchers conflate the two and classify context-sensitivity as a form of tolerance, or partial invariance.

In theory, even a completely context-specific code could produce false conclusions of invariance in neuroimaging decoding studies, due to the transformation produced by the measurement model (see Fig 1C). To provide evidence for such a general claim, we turn to simulation and theoretical work (for details on the models and procedures used in the simulations, see *Simulations* in the Materials and methods section). We study a case of complete context-specificity in which it cannot be claimed that any amount of tolerance exists in the neural representations.

**Fig 7:**
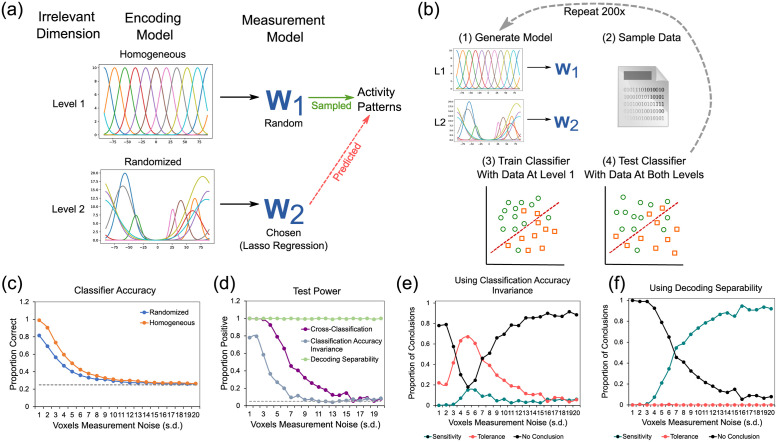
Description and results of simulation 1. A: Encoding models used in simulation 1. B: Steps taken in each repetition of simulation 1. See main text for details. C: Classifier accuracy scores for model-generated data from both levels of the context dimension. The *y*-axis represents accuracy scores, the *x*-axis represents level of measurement noise (in units of standard deviation), the dotted line represents chance performance. D: Proportion of positive tests of each type. The *y*-axis represents proportion of positives, the *x*-axis represents measurement noise, the dotted line represents the accepted false discovery rate of 5%. Panels E-F show the proportion of each type of conclusion in Table 1 (specificity/sensitivity in red, invariance/tolerance in blue, and no conclusion in green) reached from jointly testing against specificity and invariance. In both cases, the cross-classification test is used against specificity. E: Conclusions reached by using the classification accuracy invariance test against invariance. F: Conclusions reached by using the decoding separability test against invariance. This figure includes public domain clipart and all other parts are original: https://freesvg.org/binary-file-vector-graphics.

To create such a model, we started by defining two sets of encoding models, corresponding to two levels of the context dimension. In context 1, the target dimension was encoded through neural channels with homogeneous features (i.e., evenly spaced position, same maximum activity, same width), as shown at the top of Fig 7A. In context 2, the target dimension was encoded through neural channels with completely randomized features, which is exemplified at the bottom of Fig 7A. Then, we produced false positive invariance by optimizing the weights of the measurement model such that the voxel-wise activity values were similar across the two levels of the context dimension (Fig 7A). Finally, we sampled data from both models and used them as input to a linear SVM classifier. As in the preceding empirical analyses, the decoder was trained on data from the first level model and tested on independent data from both the first and second level models (Fig 7B). This entire procedure was repeated 200 times per simulation run, and we present the average results across simulations. We performed twenty simulation runs, where we gradually increased the measurement noise in each voxel (standard deviation going from 1 to 20, in steps of 1).

Fig 7C shows the decoding accuracy results from this simulation. The most important values are represented by the blue curves, which represent performance of the classifier in the non-trained level of the context dimension. Whenever accuracy is above chance, represented by the dotted line, the cross-classification test leads to a conclusion of invariance in a situation where no invariance exists (i.e., false positive invariance). The cross-classification accuracy score was much higher than chance across all levels of noise, even as measurement noise was drastically increased. The purple line in Fig 7D shows the proportion of false positives for the cross-classification test, which consistently remained above the nominal *α* = .05, represented by the dotted line, across all levels of noise that produced above-chance decoding. Only when decoding accuracy drops to chance levels (a case where the test would not be applied in an empirical setting) the cross-classification test stops producing false positives.

These results suggest that a suitable selection of measurement model is sufficient for inducing false positives in the cross-classification test, even when the underlying encoding distributions themselves show absolutely no tolerance. The next question is whether using additional tests against the null of invariance can lead to more valid conclusions.

The light blue line in Fig 7D shows the proportion of tests correctly rejecting the null of invariance for the classification accuracy invariance test. The test is very sensitive to measurement noise, having good power (about 80%) only at the smallest levels of measurement noise. Fig 7D shows the proportion of each type of conclusion in Table 1 (specificity/sensitivity in teal, invariance/tolerance in red, and no conclusion in black) reached from jointly testing against specificity and invariance, by using the cross-classification and classification accuracy invariance tests, respectively. This strategy does lead to more valid conclusions at either low or high levels of noise, but at intermediate levels the strategy fails and produces a high proportion of conclusions for tolerance. Note that these intermediate levels of noise produce decoding accuracy around 40%-60%, which are realistic values for a four-alternative classification task. The reason for the increase in conclusions of tolerance at intermediate levels of noise is related to the differential sensitivity of the tests to noise. As seen in Fig 7D, the power of the classification accuracy invariance test to correctly detect evidence against invariance drops rapidly with increments in noise, whereas the power of the cross-classification test to incorrectly detect evidence against specificity drops more slowly. Thus, at intermediate levels of noise, the cross-classification test still provides false evidence against sensitivity at a high rate, whereas the more noise-sensitive classification accuracy invariance test has rapidly dropped in its ability to provide evidence against invariance. Note, however, that this is likely to be related to the specific setup of our simulation, in which a measurement model was found that increased the likelihood of false invariance at the level of measured patterns of activity.

The green line in Fig 7C shows the proportion of tests correctly rejecting the null of invariance for the decoding separability test. The first notable result is the high sensitivity of the decoding separability test to violations of invariance. At all levels of noise, the test detected such violations in almost all simulation runs. Note that the test is sensitive even when decoding accuracy has dropped to chance. All these features of the test are expected from the theory used to develop it [4]. Higher sensitivity than accuracy-based tests is expected because the test uses information from the full distribution of decision variables from the decoder. Robustness in the face of measurement noise is expected because although noise reduces high-frequency differences between distributions, it preserves differences at lower frequencies (see [4]). We must note that this simulation probably over-estimates the test’s sensitivity, as our experimental results showed that the test often misses significance in real data.

Fig 7F shows the proportion of each type of conclusion in Table 1 (specificity/sensitivity in teal, invariance/tolerance in red, and no conclusion in black) reached from jointly testing against specificity and invariance, by using the cross-classification and decoding separability tests, respectively. In this case, invalid conclusions of tolerance are never reached. Counterintuitively, valid conclusions of sensitivity increase over inconclusive results as noise increases. The reason is that cross-classification is more sensitive to noise than decoding separability.

Overall, the results from this simulation provide further evidence favoring our hypotheses, showing that cross-classification can lead to false positive conclusions of tolerance when absolutely no tolerance exists in the underlying neural code, and that the addition of tests against invariance leads to more valid conclusions. The results suggest that decoding separability should be preferred over classification accuracy invariance to test against invariance, as was expected from theory [4].

#### Evaluating the pervasiveness of the false positive invariance problem

A critical reader might argue that the conditions leading to false positive invariance in the first simulation, namely the explicit selection of the measurement weights that produce similar voxel-wise activity patterns across levels of the context dimension, are unlikely to occur in real fMRI experiments. The true measurement process is not trained to make activity values similar across different levels of irrelevant dimensions. How pervasive is the false positive invariance problem uncovered in the first simulation? Here we show that, against intuition, the problem is quite pervasive.

In the standard encoding model used in our simulations, the mean response of neural channel *c* to stimulus *s*_*i*_, presented in context *j*, is given by a tuning function *f*_*jc*_(*s*_*i*_) (see subsection Model in Materials and methods) We can collect the mean response of *N*_*c*_ channels in a population response vector $f_{j}\left(s_{i}\right)=[f_{j1}\left(s_{i}\right),f_{j2}\left(s_{i}\right),\ldots f_{jN_{c}}\left(s_{i}\right)]$. A number of stimulus values for the target dimension are presented in any experiment, indexed by *i* = 1, 2, …, *N*_*s*_. Without loss of generality, we can focus on an experiment with two stimulus contexts indexed by *j* = 1, 2, as in our simulation. The measured activity in voxel *k* to stimulus *s*_*i*_ in the first context is equal to **f**_1_(*s*_*i*_)^T^**w**_*k*1_, and in the second context is equal to **f**_2_(*s*_*i*_)^T^**w**_*k*2_. The measurement vectors **w**_*k*1_ and **w**_*k*2_ produce invariance in voxel *k* when they produce the same mean activity value:

$$\begin{array}{ccc} 0 & = & f_{1}{\left(s_{i}\right)}^{\text{T}}w_{k1}-f_{2}{\left(s_{i}\right)}^{\text{T}}w_{k2}\\ 0 & = & {\left[\begin{array}{c} f_{1}\left(s_{i}\right)\\ -f_{2}\left(s_{i}\right) \end{array}\right]}^{\text{T}}[\begin{array}{c} w_{k1}\\ w_{k2} \end{array}]\\ 0 & = & f_{+}\left(s_{i}\right)w_{k+}^{}, \end{array}$$

where **f**_+_(*s*_*i*_) is a row vector of concatenated mean population responses, and **w**_*k*+_ is a column vector of concatenated weights.

If we collect the vectors **f**_+_(*s*_*i*_) in response to the experimental stimuli in a matrix:

$$\begin{array}{c} F_{+}=[\begin{array}{c} f_{+}\left(s_{1}\right)\\ f_{+}\left(s_{2}\right)\\ \ldots \\ f_{+}\left(s_{N}\right) \end{array}], \end{array}$$

we get a set of homogeneous equations that can be solved for **w**_*k*+_ ≠ 0:

$$\begin{array}{ccc} 0 & = & F_{+}w_{k+} \end{array}$$ (1)

A measurement model produces false positive invariance when **w**_*k*+_ ≠ **0** is a solution of this equation for all voxels *k*. Another way to see this equation is that **w**_*k*+_ corresponds to the nullspace of matrix **F**_+_. The *nullity-rank theorem* tells us that the dimensionality of this nullspace, or nullity, equals the number of columns in **F**_+_ (i.e., the total number of channels in the model) minus its rank. The nullity gives us information about the size of the subspace of measurement models **w**_*k*+_ that produce false positive invariance. When the only solution for Eq 1 is the trivial solution **w**_*k+*_ = **0**, the nullity of **F**_+_ is zero. In this case, constraints in the encoding model and experimental design, summarized in **F**_+_, are such that there is no measurement model that can produce false positive invariance. This is the *only* case in which we would not have to worry about false positive invariance, but it has been the default assumption of researchers applying the cross-classification test in the literature. Note also that this analysis is only concerned with *strict invariance* and not with *tolerance*; even when false positive invariance cannot be produced by a measurement model, false positive tolerance may still be possible.

**Fig 8:**
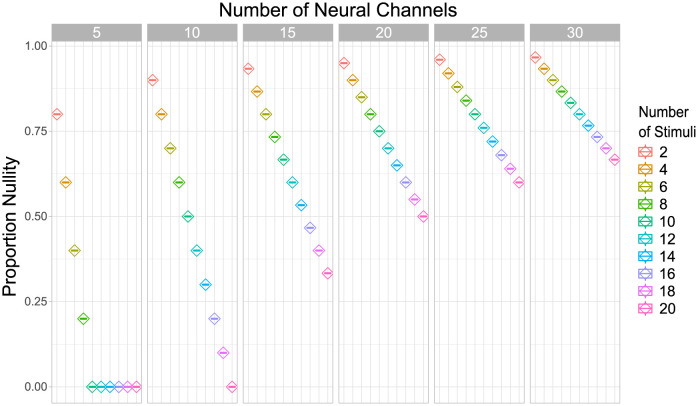
Pervasiveness of the problem of false positive invariance for the extreme case of context-specificity studied in simulation 1. Proportion nullity represents the proportion of all dimensions in the measurement space that would produce false positive invariance, and therefore the size of the false positive invariance problem. The values reported were always the same for a given combination of number of neural channels and number of stimuli, across 200 randomly sampled encoding models.

We are now in a good position to evaluate the pervasiveness of false positive invariance in the encoding scenario posed by our first simulation. We created encoding models just as indicated for simulation 1 (see Fig 7A), each time with a different number of stimuli and neural channels. The number of neural channels was varied from 5 to 30 in steps of 5, and the number of stimuli was varied from 2 to 20 in steps of 2. For each combination of neural channels and stimuli, we created 200 different encoding models, and computed the nullity of the mean population response matrix **F**_+_. As indicated earlier, the nullity represents the dimensionality of the subspace of measurement models that would produce false positive invariance. To ease comparison, Fig 8 shows the nullity divided by the dimensionality of the measurement model, or proportion nullity. This represents the proportion of the measurement space (in terms of dimensionality) that would produce false positive invariance. We found that there was no variability of results across the 200 sampled models, so Fig 8 shows the unique value of proportion nullity found in each case.

One can easily see from Fig 8 that the scenario posed in our first simulation is far from rare. On the contrary, with ten channels and four stimuli, as we used in that simulation, the proportion nullity is 0.8, meaning that the large majority of the possible measurement models will lead to false positive invariance. This result was not idiosyncratic to the parameters chosen for our simulation, with proportion nullity in general being quite high. The exception was a combination of a high number of stimuli and low number of channels, which is rare in experiments reported in the literature. Most neuroimaging studies using cross-classification to study invariance have presented 2–4 stimuli, a case in which the proportion nullity is at least 0.6, and in most cases above 0.8.

We must remind the reader that we are studying here an extreme case of context-specificity, under the assumption of no measurement noise, and an extreme case of false positive invariance, rather than tolerance. For these reasons, we can consider our results a lower bound on the size of the false positive invariance problem. More realistic scenarios involving context-sensitivity, high measurement noise, or evaluation of tolerance rather than strict invariance can all be expected to worsen the problem beyond what is shown in our results.

We see two clear trends in Fig 8. First, proportion nullity –and therefore, the problem of false positive invariance– drops linearly with number of stimuli included in the study. Experimenters can reduce the risk of false positive invariance by increasing the number of stimulus levels for the target dimension. Second, proportion nullity increases in a negatively accelerated fashion with increments in the number of neural channels. The number of neural channels represents our assumption of how many unique neural tuning functions underlie the data or, in other words, how well-covered is the stimulus dimension by the encoding neural population. In realistic scenarios, this value will be much higher than any of those shown in Fig 8. However, it is common to find applications of the standard encoding model in computational neuroimaging that assume 6–15 channels (e.g., [19–22]).

#### Simulation 2: False positive invariance resulting from similarly tuned neural subpopulations across contexts

A critical reader may again argue against the results just presented, indicating that although the space of possible measurement models leading to false positive invariance is large in most published studies, most of those models would never be observed in nature. Only a small proportion of all possible measurement models might be truly at play in neuroimaging studies, and those could be contained within the space of models for which false positive invariance is not an issue. Although this is an extremely optimistic position, and we think that it would be unwise for scientists to take it, we would like to strengthen our conclusions by studying a realistic encoding scenario, likely to be implemented in the brain.

There are many known cases in which neurons that are sensitive to a particular stimulus feature are spatially clustered at sub-millimeter scales. In those cases, while there is spatially distributed information about stimulus features, this information is not immediately visible at the typical resolution of an fMRI study. For example, V1 neurons that are sensitive to the same spatial frequency, color, ocular dominance, and orientation all cluster at the sub-millimeter scale [23–26]. Although advances in high-field fMRI can in some cases uncover such sub-millimeter organization (e.g., [27]), information can also be spatially distributed without any clustering (e.g., “salt-and-pepper” codes; see [28, 29]), at scales that are unlikely to be reached with fMRI at even higher field strengths than those currently available [30, 31].

In cases such as these, across voxels we would expect to find relatively homogeneous distributions of selectivities. Our ability to use voxel-level decoding to detect whether and how features are encoded depends critically on small random variations in mixing; that is, in the proportion of each type of neuron present within each voxel. Indeed, small differences in mixing across voxels is a mechanism proposed to underlie decoding of orientation information from V1 [32–36], like that shown in our experimental study.

This sub-voxel distribution of information, which may underlie the success of many fMRI decoding studies, can also easily lead to false-positive invariance when the cross-classification test (or other tests of the null of specificity) is used in isolation. Small differences in mixing might be enough to promote above-chance decoding of a stimulus feature, because decoding algorithms are specifically trained to detect differences in the target feature. On the other hand, decoding algorithms are not trained to detect changes in context. Any small differences in mixing that might provide information about context-specificity would be lost, and the decoding algorithm would be very likely to generalize performance across changes in stimulus context. We find an example of this in our own experiment. There, classification of spatial position generalized perfectly across changes in grating orientation, as shown in Fig 3, despite the fact that the voxels contained information about differences in orientation, as determined by above-chance decoding of that dimension (see Fig 4).

**Fig 9:**
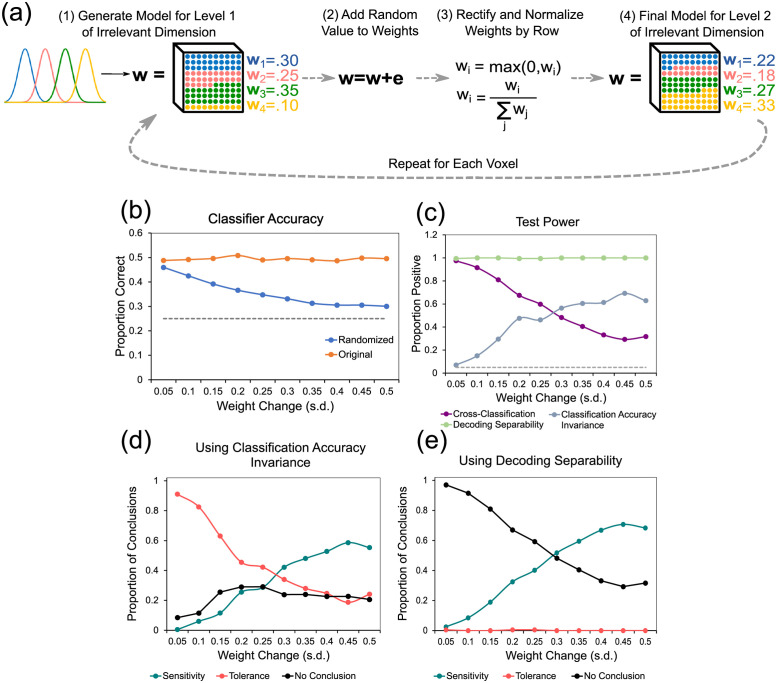
Description and results of simulation 2. A: Encoding model for simulation 2. See main text for details. Panels B-E show the decoding results from simulation 2. B: Classifier accuracy scores for model-generated data from both levels of the context dimension. The *y*-axis represents accuracy scores, the *x*-axis represents magnitude of noise added to measurement weights for the second level model, the dotted line is chance performance. Proportion of positive tests of each type. The *y*-axis represents proportion of positives, the *x*-axis represents measurement noise, the dotted line represents the accepted false discovery rate of 5%. Panels C-D show the proportion of each type of conclusion in Table 1 (specificity/sensitivity in red, invariance/tolerance in blue, and no conclusion in green) reached from jointly testing against specificity and invariance. In both cases, the cross-classification test is used against specificity. D: Conclusions reached by using the classification accuracy invariance test against invariance. E: Conclusions reached by using the decoding separability test against invariance.

In the present simulation, we wanted to study the sensitivity of different fMRI decoding tests to changes in mixing carrying information about context-sensitivity. With this goal in mind, we created a model in which a target dimension is encoded in a completely context-specific manner, with one subpopulation of neurons responding whenever the context dimension is at level 1, and a different subpopulation of neurons responding whenever the context dimension is at level 2. Both subpopulations were modeled using a standard homogeneous encoding model (see above), but note that this similarity in tuning functions is not equivalent to invariance, as each channel responded *only* at one of the levels of the context dimension. In other words, our simulation assumes that populations encoding the target dimension are completely separated across levels of the context dimension, but they encode the target dimension in a similar way (just as neurons in Fig 1D have two selectivity types across levels of the context dimension). As before, we report the averaged results from 200 simulations in each run. Measurement noise was set to a fixed level across simulations (s.d. = 5, which in our previous simulation produced accuracies around 40%-50%, see Fig 7C). In each simulation run, we increased the difference in the measurement models for the two levels of the context dimension, by adding random noise to weights of the measurement model as illustrated in Fig 9A. The standard deviation of the weight noise was gradually increased from 0.05 to 0.5 (i.e., from 0.5 to 5 times the average weight value), in steps of 0.05. That is, in the final models the contribution of each neuron type (e.g., neurons selective to a value of 0 in the target dimension) was widely different across levels of the context dimension.

The results from this simulation are shown in Fig 9C–9E. Panel B shows the accuracy of the classifier tested in the original training context (red line) and in the changed context (i.e., cross-classification performance; blue line). It can be seen that the cross-classification test is sensitive to mixing variations, as accuracy drops with increments in weight changes with context. However, accuracy remains well above chance even for the largest weight changes. Fig 9C shows the proportion of positive tests as a function of the magnitude of random weight changes (in standard deviations). The cross-classification test consistently showed false positives at a rate much higher than the nominal 5%. High levels of false-positive invariance were present even when the weight noise standard deviation was five times as large as the average weight values. These results suggest that, when two *completely separate* neural populations use similar codes to represent a target dimension across levels of an context dimension, false positive invariance is likely to be found not only with the small variations in mixing that one would usually expect from fMRI studies, but from very large variations in mixing.

As before, the question now is whether this issue of false-positive invariance can be ameliorated by adding tests against the null of invariance. Using classification accuracy invariance, the results are not very promising. The light blue line in Fig 9C shows the power of this test to reject the null of invariance, which starts near zero with very small variations in weights (or mixing) and is quite low (about 60% power) even at the largest weight variations. Fig 9D shows the proportion of each type of conclusion in Table 1 (specificity/sensitivity in teal, invariance/tolerance in red, and no conclusion in black) reached from jointly testing against specificity and invariance, by using the cross-classification and classification accuracy invariance tests, respectively. First, the addition of classification accuracy invariance does improve the validity of conclusions. Comparing the purple curve in Fig 9C against the red curve in Fig 9D shows that the latter drops more steeply with size of weight changes. On the other hand, using cross-classification and classification accuracy invariance together still leads to a false positive rate above 5% across all the values of weight change simulated.

On the other hand, using decoding separability the results are much better. The green line in Fig 9C shows that the power of this test to reject the null of invariance is near 100% across all levels of weight change. That is, the test is sensitive to even small changes in mixing resulting from changes in context. As explained before, this high power is a consequence of the test using the whole distribution of decision variables from the decoder, rather than only binary classification decisions. Fig 9E shows the proportion of each type of conclusion in Table 1 (specificity/sensitivity in teal, invariance/tolerance in red, and no conclusion in black) reached from jointly testing against specificity and invariance, by using the cross-classification and decoding separability tests, respectively. First, the test has a higher power than classification accuracy invariance to reach the correct conclusion of context-sensitivity. More importantly, at low values of mixing, the test leads to inconclusive results rather than to the incorrect conclusion of invariance.

Overall, this simulation confirms our previous conclusion and theoretical expectation that supplementing the cross-classification test with a test against the null of invariance increases the validity of conclusions about the underlying codes, and that decoding separability is superior to classification accuracy invariance for that goal. We have shown that this is the case in the realistic scenario in which two different populations encode the target dimension in a similar manner, and the fact that both populations are separated can be inferred only from small differences in their relative contribution to voxel activities. With such small differences in mixing (i.e., the smallest values of weight change in Fig 9A), using cross-classification alone leads to a conclusion of false positive invariance almost 100% of the time, the addition of the classification accuracy invariance test slightly reduces the issue, and the addition of the decoding separability test eliminates it, at least in our simulation. The results were similar at very large differences in mixing (i.e., the largest values of weight change in Fig 9A), where using cross-classification alone leads to a conclusion of false positive invariance about 30% of the time, the addition of the classification accuracy invariance test slightly reduces the issue, and the addition of the decoding separability test eliminates it, with the most likely conclusion being the ground truth of context-sensitive encoding. We must warn again, however, that the sensitivity of the decoding separability test is expected to be lower with experimental data, as it was in our own study. The main reason why decoding separability is so extremely powerful in our simulations is that they assumed the extreme case of completely context-specific codes.

#### Simulation 3: On the difficulty to obtain a valid continuous measure of invariance/specificity

As mentioned in the introduction and illustrated in Fig 1A, neural representations are likely to vary along a continuum between complete context invariance and specificity. Given this, it might be surprising to the reader that we advocate using inferential tests against the extremes of this continuum rather than proposing a single continuous measure of invariance/specificity. We believe that the influence of the measurement model makes it difficult to obtain a measure that is valid and precise, in the sense of providing information of exactly where in the continuum the underlying neural representations lie.

**Fig 10:**
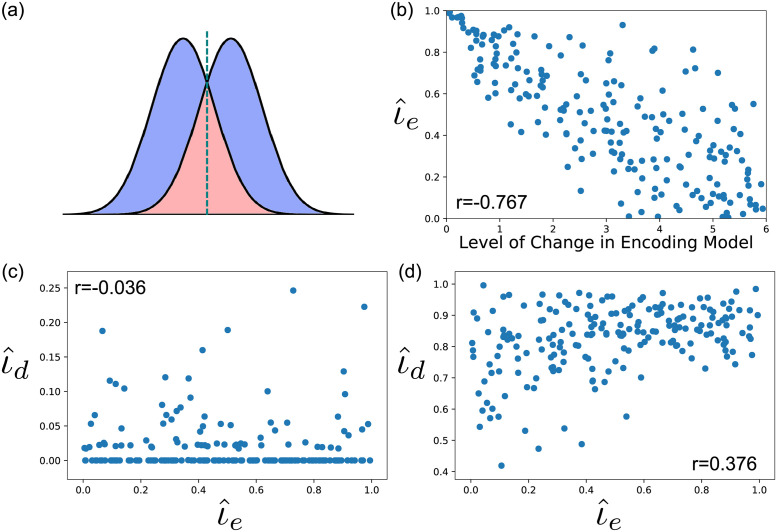
Description and results of simulation 3. A: Illustration of the *L*1 (blue) and AC (red) distances between two distributions. The green dotted line represents the optimal classification bound (i.e., the value with equal likelihood to belong to either distribution). The proposed invariance coefficient represents the proportion of overlap between the two distributions (red area over the sum of red and blue areas). B: Correlation between the continuous level of change implemented in the encoding model and ${\overset{⌢}{\iota }}_{e}$. C-D: Construct validity of the two versions of ${\overset{⌢}{\iota }}_{d}$ (i.e., their correlation with the true value ${\overset{⌢}{\iota }}_{e}$): one version computed by decoding stimulus values (panel C) and another by decoding level of context (panel D).

To illustrate this point, here we propose a new measure of invariance/specificity–the invariance coefficient or *ι*–, show that this measure can provide information about continuous changes in invariance/specificity when computed directly on representations at the neural encoding level but it does a relatively poor job retaining that information when computed using decoding distributions obtained from indirect measures of neural activity. The measure, and ways to estimate it from multivariate activity patterns, is described in detail in the Materials and methods section (subsection Simulation 3: On the difficulty to obtain a valid continuous measure of invariance/specificity). It takes two probabilistic representations of the same stimulus in different contexts, and computes their proportion of overlap. Another interpretation of the measure is the ratio of how confusable over how discriminable the two representations are. To understand why, note that the optimal strategy to classify a random sample *X* as belonging to one distribution or the other is to choose the distribution for which *X* has highest likelihood. An example of an optimal classification bound is given by the teal dotted line in Fig 10 (in a multidimensional space this bound is not required to be a line). With this in mind, and assuming that the distributions are approximately symmetric, note that *ι* corresponds to the ratio of the sum (across the two distributions) of the probabilities of incorrect classification (red area in Fig 10) over the sum of probabilities of correct classification (blue plus red areas in Fig 10). Note that here we are referring to classification of a random vector or variable as presented in context 1 or 2, or *context decoding* rather than *stimulus decoding*, which is the focus in the rest of our manuscript and in the literature at large. When *ι* = 0, the two distributions are perfectly discriminable, a case of extreme context-specificity. When *ι* = 1, the two distributions are perfectly confusable, a case of context-invariance. Values between these extremes provide a continuous and interpretable measure of context-tolerance (or its inverse, context-sensitivity).

We can estimate this measure for representations at the level of neural encoding, represented by ${\overset{⌢}{\iota }}_{e}$, and also for decision variables at the level of decoding from indirect measures of activity, represented by ${\overset{⌢}{\iota }}_{d}$. Importantly, ${\overset{⌢}{\iota }}_{d}$ can be computed from values obtained from a number of decoders. In line with the rest of this work and the literature at large, we computed a first version of ${\overset{⌢}{\iota }}_{d}$ using a support vector classifier trained to decode the *stimulus* value in the target dimension. More in line with our theoretical interpretation of the index, we computed a second version of ${\hat{\iota }}_{d}$ using a support vector classifier trained to decode the *context* in which the stimulus was presented. We reasoned that focusing on discrimination of contexts rather than stimuli would produce a more valid measure, in the sense of reflecting the true underlying level of invariance/specificity as measured at the level of encoding.

We performed a simulation in which the encoding model properties were continuously modified from complete invariance to complete specificity (for a description of methods; see subsection Simulation 3: On the difficulty to obtain a valid continuous measure of invariance/specificity in the Materials and methods section). The simulation was repeated 200 times, each time randomly choosing the level of invariance/specificity of the encoding model. Our goal was to answer the following questions: How well does ${\hat{\iota }}_{e}$ capture continuous variation in invariance/specificity built into the encoding model? How well do the two versions of ${\hat{\iota }}_{d}$ capture the true variability in invariance as measured from neural representations at the level of encoding (i.e., what is their construct validity)?

The results of this simulation are presented in Fig 10. Fig 10B shows the correlation between the continuous level of change implemented in the encoding model and the invariance coefficient computed at the level of encoding. The correlation was quite high at -0.767, *p*<.001, and the relation between the variables seems linear. Thus, when computed at the level of neural encoding, the invariance coefficient can capture continuous changes in invariance implemented in the encoding model.

Fig 10C and 10D show the correlation between the two versions of the decoding invariance coefficient ${\hat{\iota }}_{d}$ and the encoding invariance coefficient ${\hat{\iota }}_{e}$. In line with our interpretation, the measure based on stimulus decoding has no construct validity with a correlation with the true measure of -0.036, *p*>.1, whereas the measure based on context decoding has a moderate construct validity with a correlation with the true measure of 0.376, *p*<.001. We conclude that the best way to compute this specific measure is by focusing on decoding of the context in which a stimulus is presented, which should be done after it has been determined that information about the target dimension is available in a brain region via a traditional stimulus decoding analysis.

With that being said, even the better version of ${\hat{\iota }}_{d}$ clearly has validity issues, as it can capture only 14.17% of the variability in invariance measured in the underlying neural representations. Fig 10B shows that a major issue with ${\hat{\iota }}_{d}$ is that it overestimates invariance, having a range between 0.4 and 1.0, whereas the true values range from 0.0 to 1.0. In addition, high values of invariance (>0.8) are estimated across the whole range of values of ${\hat{\iota }}_{e}$. This is of course a consequence of the loss of information imposed by the measurement model, and particularly the ability of the model to induce false invariance already discussed. Specific features of the measurement model are likely to influence the measure’s construct validity. Our goal here was not to explore all these possibilities, but rather to present the measure and illustrate how the measurement model limits our ability to precisely measure invariance. We believe that issues with validity are likely to arise from any other index computed from indirect measurements of neural activity.

## Discussion

Here, we have provided empirical and computational evidence supporting two insights about decoding tests of invariance reached with the help of neurocomputational theory [4]. First, that tests aimed at evaluating evidence against the null of context-specificity, and for the alternative of context-invariance, may be prone to false positives due to the way in which the underlying neural representations are transformed into measurements. Second, that jointly performing tests against the nulls of invariance and specificity allows one to reach more precise and valid conclusions about the underlying representations.

In the empirical study, we performed decoding of orientation and spatial position from fMRI activity patterns recorded in V1, a case in which properties of the underlying neural code are known. The cross-classification test gave strong evidence for the incorrect conclusion that, in V1, encoding of spatial position is tolerant/invariant to changes in orientation, as well as some evidence for the incorrect conclusion that orientation is tolerant/invariant to changes in spatial position. We found that the addition of theoretically-derived tests of invariance leads to more valid conclusions regarding the underlying code.

The results of two simulations strengthened the conclusions from the empirical study, by showing that they hold even in the extreme case of completely context-specific encoding. In the first simulation, we showed that cross-classification can lead to false positive conclusions of tolerance when absolutely no tolerance exists in the underlying neural code, and that the addition of tests against invariance leads to more valid conclusions. We also showed, through theoretical analysis and further simulations, that this problem is likely to be pervasive, rather than resulting from a hand-picked proof of concept. In our second simulation, we showed that the same results are found in simulations of realistic encoding scenarios.

Based on our empirical and computational results, we conclude that the cross-classification test can lead to invalid conclusions about the invariance of neural representations. Applying the test by itself should be avoided, and previous research using the test should be re-evaluated in light of our results. Instead, we propose to routinely test against the null of invariance whenever the cross-classification test is applied. Even if a researcher is unconvinced by the pervasiveness of the problem highlighted in our study and simulations, the cost of running these additional tests is extremely low.

Note that we have purposefully relied on our own data and simulations to make the point that using the cross-classification test alone can lead to invalid conclusions about invariance. This is because our intention is not to single-out specific studies that have used cross-classification in the past, but rather to alert users of this test, and the neuroscientific community at large, that its results should be taken with caution until further tests against context-invariance or evidence from direct measurements of neural activity are also available. We believe that the problems with cross-classification highlighted here are serious enough that researchers should re-evaluate all published claims of invariance stemming from this test in light of new analyses and/or data.

As expected from theory, we found that the decoding separability test is sensitive to violations of invariance that cannot be captured by the classification accuracy invariance test. In particular, when decoding accuracy is near ceiling or floor values, only the decoding separability test can detect violations of invariance by relying on the more fine-grained information available in the full decoding probability distributions, rather than on the coarse information available in accuracy estimates. The reason behind this superiority is simple: the decoding separability test uses information from the full distribution of decoder decision variables, and much of this information is lost once that distribution is binarized for classification. A similar conclusion was reached by Walther et al. [37], who found that the reliability of continuous neural dissimilarity measures was higher than that of classification accuracies, and concluded that this was due to the loss of information inherent to the latter. We believe that focusing on full decoding distributions can help us to move from using decoding to test *whether* information is encoded in a particular area, to using decoding to test *how* information is encoded. Additional examples of this approach have linked uncertainty in decoding distributions to behavior [38], and have correlated the variability in decoding distributions to behavioral responses [39].

We also showed through simulation that the measurement transformation limits our ability to obtain a valid continuous index of invariance/specificity when computed from indirect measures of neural activity. Even when computed under ideal circumstances (small changes imposed by the measurement model and 200 presentations of each stimulus), the decoding invariance coefficient could capture only 14.17% of the variability in invariance measured in the underlying neural representations. While we did not explore all possibilities, our simulation results help us make the point that precise measurement of invariance/specificity from neuroimaging data is a difficult task.

### Alternative measures of neural activity and data analysis techniques

Because our empirical study involved fMRI, we have framed the discussion here mostly in terms of that neuroimaging technique. Note, however, that our theoretical and simulation results hold for studies using *any* indirect or aggregate measure of neural activity, which includes M/EEG, ECoG, and LFPs, among others. Indeed, the linearized measurement model common in the fMRI literature and used in our simulations is also commonly used in those other techniques (e.g., [40–42]). Of course, the level of aggregation and spatial specificity of a technique will modify the gravity of the problem for any specific technique. However, as noted earlier, information can be spatially distributed in the brain without any clustering [28, 29] and thus the issue of false positive invariance may be present to a certain degree with any aggregate indirect measure of neural activity.

Similarly, our conclusions are unlikely to be limited only to decoding data analyses. Other approaches, such as forward and inverted encoding modeling (for reviews, see [43, 44]) and representational similarity analysis [45] are likely to suffer from similar issues. The reason is that the possibility of artificially increasing the appearance of invariant representations is inherent to the transformation from the neural space to the measurement space (see Fig 1b). That is, the problem lies within the measured patterns of activity themselves, rather than with the analyses performed over those activity patterns. Any analysis aimed to obtain evidence of invariance is likely to be based on the observation of high similarity of activity patterns across changes in context, and that similarity can be artificially increased by the measurement model.

That being said, analysis methods such as inverted encoding modeling have been developed to provide evidence of changes in neural representation that result from changes in some experimental factor, naturally lending themselves to the detection of evidence for context specificity (i.e., against invariance). Inverted encoding modeling has indeed been applied in such a way (e.g., [46]), and this application can be considered analogous to the decoding tests against invariance studied here, rather than to the more problematic cross-classification test. However, inverted encoding modeling is a parametric approach and as such it can fail to yield valid conclusions when the assumptions of the model are incorrect (e.g., wrong selection of tuning functions; see [47]). On the other hand, tests based on decoding are nonparametric, yielding valid conclusions without making any assumptions about encoding or measurement (see [4]).

The decoding separability test advocated here is based on the calculation of a distance measure. Specifically, the absolute distance (i.e., the *L*1 distance) between distributions of decision variables obtained from a linear classifier. A large number of other distance measures have been used and evaluated in representational similarity analysis [37, 48], and it is not clear to what extent such measures might provide evidence against invariance that is as good or better than the *L*1 distance. Preliminary results of simulation studies suggest that distance measures vary widely in their construct validity; that is, their ability to reflect the true underlying distances between neural representations at the level of encoding [49]. The *L*1 measure is among those with highest construct validity, but other good measures according to this criterion are the inner product, Mahalanobis, and euclidean distances. We prefer the *L*1 distance in the decoding separability test mainly because we have previously shown that the use of this distance allows the test to provide valid inferences about underlying deviations from invariance at the level of neural encoding [4]. Similar proofs have not been provided for other measures, which is not to say that they are not possible. The Mahalanobis distance is of particular interest, as it has been shown to provide superior reliability in previous studies [37]. A disadvantage of this measure is that it computes a distance between distributions based solely on their mean and variance, implicitly assuming that those distributions are multivariate normal. On the other hand, the *L*1-based decoding separability test does not need to make any assumptions about the distribution of the data, besides assuming that measurement error is additive, and it can detect differences between distributions in higher-order moments that are impossible to detect using Mahalanobis (see discussion on the multivariate general linear model in [4]).

### Recommendations for researchers

Our results have consistently shown that there is an inherent disadvantage with tests aimed at providing evidence for invariance (or rather, against context-specificity), which tend to yield false positives. However, findings of invariant representation are interesting to many neuroscientists and they could inform theories of neural processing. We recommend those researchers both to use the double-test strategy developed here and to be extremely cautious regarding conclusions of invariance obtained from any indirect or aggregated measures of neural activity (fMRI, EEG, ECoG, LFPs, etc.), which should be held as tentative until evidence from direct measurements of neural activity are available.

The same is not true about tests aimed at providing evidence for context specificity (or rather, against context-invariance): in this case, the tests are much more likely to yield valid conclusions, and no protection is needed against false positives. But note that a positive test against context invariance only provides evidence for the valid inference that the underlying representations are not invariant. The representation can still be anywhere else in the continuum depicted in Fig 1a. Therefore, the two-test strategy would still provide additional evidence regarding the underlying representations, at the quite low cost of running one additional test on the same data.

In sum, we recommend all researchers to use a two-test strategy when attempting to make inferences about invariance/specificity from indirect measures of neural activity, and to interpret the results of the tests using Table 1. We also recommend to use the decoding separability test to test against the null of invariance. In theory, this test can capture evidence against invariance that is not available from the classification accuracy invariance test, and here we found that this is indeed the case in some real-world examples (e.g., decoding of spatial position across changes in orientation from V1).

That being said, researchers should understand that application of the decoding separability test is most useful with large datasets that ensure accurate estimation of full decoding distributions. Our empirical results were obtained using a design in which a large dataset was obtained from each participant. We recommend that researchers use the same kind of design. Most datasets used in the past to study invariance do not fit that description, instead having a small number of stimulus presentations and/or not having explicitly separate datasets for training and testing. We expect that researchers will be faced with such non-ideal datasets, either because they want to reanalyze data obtained in the past or because budgetary considerations limit the number of stimulus presentations that can be included in a study. To deal with such suboptimal datasets, researchers can use the classification accuracy invariance test against the null of invariance, which requires a smaller number of stimulus presentations than the decoding separability test, because it calls for the estimation of single values (accuracies) rather than the estimation of whole densities. In addition, the dataset can be used more efficiently by estimating accuracies through leave-one-out cross-validation.

Our results regarding the pervasiveness of the false positive invariance problem (Fig 8) show that this problem can be greatly reduced by including a large number of stimuli spanning the stimulus dimension under study. This is an important theoretical insight, as number of unique stimulus values is a design factor under the researcher’s control. We recommend that researchers use as many unique stimulus values as possible in their designs.

### Limitations and future work

During a study with a large number of trials the neural representations under study might themselves change. Factors such as fatigue, adaptation, and familiarization could all produce changes in brain representation. Because our interest is not on all aspects of neural representation, but only on context specificity/invariance, the key question here is whether a long experiment would produce changes in the neural representation of a stimulus that are specific to one context and not others. We believe that this is unlikely to be the case in any study with two features. First, a balanced design, in which each stimulus is presented equally often in different contexts across the whole experiment. With a balanced design, factors such as fatigue, adaptation, and familiarization should influence the stimulus representation equally across contexts. The influence of some of those factors may also be reduced by dividing the study into multiple short sessions separated by long intervals (e.g., one day or more). Second, when a behavioral task is used to keep participants’ attention on the stimuli, the task should be unrelated to the stimulus features under study, to avoid potential adaptive changes in representation due to learning, selective attention, etc. The study presented here satisfies both of these criteria and therefore we believe that its length was unlikely to bias the results.

Another potential limitation of our empirical study has to do with segmentation of V1, which in our study was carried out using an algorithm [50] that focuses on the analysis of cortical folds, rather than on the results of a functional localizer. The evidence shows that this algorithm has a precision that is equivalent to up to 25 minutes of functional mapping. Thus, we could have performed a functional localizer longer than 25 minutes in order to increase the precision of V1 segmentation. We did not see value in that approach, as the neighboring area V2 is also retinotopic (spatial position and size of receptive fields is similar for V1 and V2 near their border) and encodes orientation similarly to V1, meaning that our predictions for V2 would be similar to those for V1.

**Fig 11:**
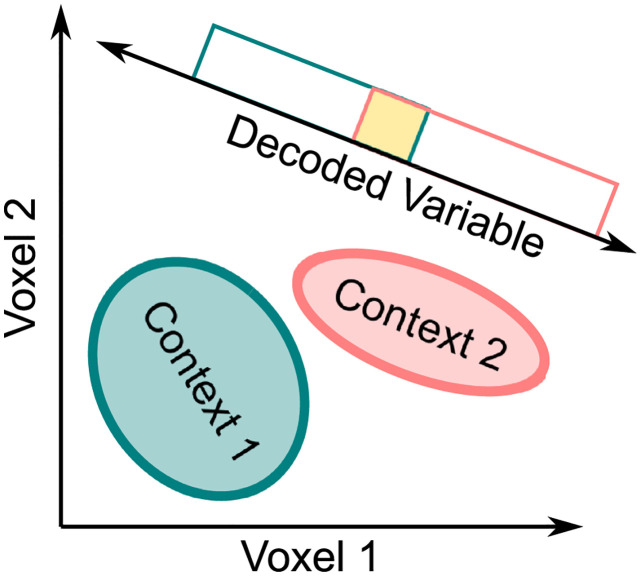
Decoding distributions cannot be used to obtain a valid test of no overlap between neural representations across two contexts. The main axes represent measurements at the voxel level, and each ellipse represents the distribution of neural activity (after transformation by the measurement model) for a target stimulus property presented in two different contexts. The two distributions are completely non-overlapping at the level of the multivariate voxel patterns. However, when the two distributions are projected onto the decoded variable, they show a non-zero overlap represented by the yellow area.

The study of invariance could benefit from the development of a test against context-specificity to replace the cross-classification test, which is relatively insensitive due to its reliance on decoding accuracy. This new test should aim to evaluate the null hypothesis that the neural representation of a target stimulus property is completely different in two different contexts, showing non-overlapping distributions of neural activity. The development and validation of such a measure is not trivial, and we must leave it to future research. We at least know that a sensitive measure would rely on something different from the decoding distribution of the target variable, and therefore it would follow a different logic than the decoding separability test developed in previous work [4]. Fig 11 shows why this is the case. The two main axes represent measurements in two different voxels, and each ellipse represents the distribution of voxel activity patterns for a target stimulus property presented in two different contexts. It can be seen that the two distributions are completely non-overlapping in the multivariate space of voxel patterns. However, when the two distributions are projected onto the decoded variable they show a non-zero overlap, represented by the yellow rectangular area. Note how changing the direction of the decoded variable does not necessarily result in no overlap. Also, simply measuring the overlap at the voxel level ameliorates but does not solve the issue, because the measurement model may also artificially introduce overlap in the distributions (Fig 1C).

An important question left open is whether tests against the null of invariance might be prone to false positives, as is the case for tests against the null of specificity. Within the theoretical framework adopted here, the answer is “no”, as in theory it is impossible for any measurement model to transform invariant representations into non-invariant activity patterns (see Fig 1C, top panel). However, in fMRI studies, activity patterns must be estimated from the BOLD response through deconvolution or other means (see Materials and methods). If changing context influences the hemodynamic response function (HRF), then this would in turn influence activity estimates, producing apparent context-sensitivity even if the underlying neural representation is fully invariant. Factors known to influence the HRF include stimulus duration [51], separate scans [52], inter-trial interval [53], stress level [54], and levels of some neurotransmitters [55–57]. Exploring which changes in the HRF could influence the results of tests against invariance is beyond the scope of this work, but researchers should design their studies so that factors known to influence the HRF do not co-vary with changes in context.

Beyond the specific case of decoding tests of invariance, the present study shows the dangers of over-reliance on operational tests that have only face validity, particularly in the study of neural representation through indirect measures obtained through neuroimaging. Our study joins other recent reports in the literature [22, 47] in showing that the application of sophisticated data analysis tools can lead to the wrong conclusions when problems of identifiability (e.g., between neural and measurement factors) inherent to neuroimaging are not taken into account. We believe that theoretical and simulation work will play an important part in the future of neuroimaging, both to point out areas in which our methods might run into issues, as well as showing us potential solutions.

## Materials and methods

### Ethics statement

This study was approved by The Social and Behavioral Institutional Review Board and by the Center for Imaging Science Steering Committee of Florida International University, and it was found to be in compliance with the institutions Federal Wide Assurance. All participants gave written consent to experimental procedures before participating in the experiment.

### Participants

Five healthy volunteers (ages 19–27, three female) from Florida International University participated in the experiment; all had normal or corrected-to-normal vision.

#### Stimuli

All stimuli were generated using Psychopy v.1.85.0 [58]. Images were displayed on a 40-inch Nordic Neurolab LCD InroomViewing Device, placed at the rear entrance of the scanner bore. Subjects viewed the screen via an angled mirror attached to the head coil. Visual stimuli were full-contrast square-wave gratings with a spatial frequency of 1.5 cycles per degree of visual angle (similar to [59–61]), a frequency known to drive V1 responses strongly [62], shown through a wedge-shaped aperture window that spanned from 1.5° to 10° of eccentricity and 100° of polar angle (Fig 2). The aperture window had four possible locations: top-right, bottom-right, top-left, and bottom-left. The square-wave gratings were oriented in one of four angles for each trial: 0°, 45°, 90°, 135°. The phase of the gratings was randomly changed every 250ms, to reduce retinal adaptation and afterimages.

### Task and procedures

To ensure that the data used to train a classifier in decoding analyses (see below) was independent from the data used to test the classifier and compute measures of performance, training trials and testing trials were presented on separate acquisition runs. Training and testing runs were identical in all aspects except one: the positions of the aperture window were restricted to top-right and bottom-left for the training runs, while testing runs included all four positions (Fig 2). During stimulus presentation, the phase of the grating was randomly shifted every 250 ms. The orientation of each grating was randomly chosen on each trial, while the spatial position of the window changed sequentially in a pre-determined manner. In training runs, the aperture window switched between top-right and bottom-left on every trial. In testing runs, the aperture window cycled through top-right, bottom-left, bottom-right, and top-left, in that order. For both training and testing runs, each combination of spatial position (two or four levels) and orientation (four levels) was presented 35 times in a single acquisition session. Each subject went through 4 identical acquisition sessions to yield a total of 135 presentations of a given combination of orientation and spatial position (see all combinations in Fig 2) for both training and testing trials types. This large longitudinal sample size (3,240 trials total per participant) was chosen to focus our analyses on data at the level of individual participants (see *Statistical analyses* below).

On each trial, a single grating was presented for 3s, followed by a 3s inter-trial interval. All runs began with a 10s fixation period and ended with a 1 min rest period. The training runs lasted for 5 mins and 43s, and the test runs lasted for 10 mins and 13s. Due to experimenter error during data acquisition, a portion of training trials were lost for participants 1 and 2. To compensate for the reduced number of training trials, we collected an additional session of data from subject 2, resulting in about 123 training trials and 112 testing trials per stimulus. For subject 1, we simply set aside half of the testing trials for training purposes and used the other half for testing; the number of testing trials for non-trained values of the context dimension remained the same as for all other participants.

The participants’ task was to look at a small black ring presented in the center of the screen (similar to [59]). The black ring had a small gap that randomly switched position throughout the trial. Participants were asked to continuously report the side of the gap (left or right) by pressing the corresponding button. The task had the purpose of forcing participants to fixate at the center of the screen, and to draw attention away from the stimuli.

### Functional imaging

Imaging was performed with a Siemens Magnetom Prisma 3T whole-body MRI system located at the Center for Imaging Science, Florida International University. A volume RF coil (transmit) and a 32-channel receive array were used to acquire both functional and anatomical images. Each subject participated in four identical MRI sessions. During each session, a high-resolution 3D anatomical T1-weighted volume (MPRAGE; TR 2.4s; TI 1.1s; TE 2.9 ms; flip angle 7°; voxel size 1×1×1 mm; FOV 256 mm; 176 sagittal slices) was obtained, which served as the reference volume to align all functional images. During the main experiment, functional images were collected using a T2*-weighted EPI sequence (TR 1.5 s; TE 30 ms; flip angle 52°; sensitivity encoding with acceleration factor of 4). We collected 60 transversal slices, with resolution of 2.4×2.4×2.4 mm, and FOV of 219mm. The first six volumes in each run were discarded to allow T1 magnetization to reach steady state.

### Statistical analyses

All data analyses, including multi-voxel decoding and tests of invariance, were performed on the individual data of each participant. In designing our experiment, we favored collection of a large amount of data per participant (3,240 trials, about 8 hours of scanning) rather than a large number of participants. Each separate analysis can be considered a replication of a single-subject experiment. With our sample sizes (*n* = 135 per stimulus), our tests can detect a 6% difference from chance in classifier performance with 85% power, an 8% drop in classifier performance with >80% power, and kernel density estimate error is maximally reduced, according to simulation studies [63].

#### Region of interest

The boundaries of V1 are commonly found using a functional localizer procedure. However, previous work has shown such boundaries can be accurately estimated from cortical folds, without the need for a functional localizer [50]. Additionally, evidence shows that the definition of V1 boundaries using the algorithm proposed by Hinds et al. [50] has a precision that is equivalent to 10–25 minutes of functional mapping [64]. Therefore, we applied the Hinds et al. [50] algorithm, implemented in Freesurfer 6.0 [65], to the anatomical T1-weighted images, to define the boundaries of V1 in each participant and obtain an ROI mask. The obtained V1 mask was then converted into a binary mask, and transformed to the individual’s functional scan space (the averaged volume of the first functional run was used as a target) using linear registration with FLIRT.

#### BOLD data preprocessing

Data were processed and analyzed using *nipype* Python wrappers for FSL [66, 67]. Basic preprocessing of functional data included skull stripping, slice time correction, and head motion correction using MCFLIRT. All functional runs for a given subject were then aligned to an averaged volume of the first functional run for the same subject. This step ensured that the entire time-series for each subject lay in the same co-ordinate space. The aligned time-series was then concatenated into a single time-series file for further processing. The concatenated series for each subject was de-trended using a Savitzky-Golay filter with a polynomial order of 3 and a window length of 81 secs [68].

#### Deconvolution

Using the obtained V1 mask, time-series from V1 voxels were extracted for further analysis. Single-trial activity estimates were obtained via a data-driven deconvolution technique in which deconvolved neural activation values and a model of the HRF are estimated together [68]. Unlike other methods that hold the shape of the HRF constant across voxels, this technique allows the shape of the HRF to be different in each voxel, resulting in more accurate activity estimates. The model is implemented via the *hrf_estimation* Python package v. 1.1 (https://pypi.org/project/hrf_estimation/). The *hrf_estimation* package presents 10 different options for HRF modeling, with varying options for the HRF basis function and for the General Linear Model estimation technique. To select the optimal combination of HRF and estimation method, we performed a cross-validated decoding analysis using data from the training runs of a single participant (data from the testing runs was not used in this pre-analysis). First, we generated activity estimates from all possible model combinations (estimation method and HRF). Then, for each model, we trained and tested an SVM classifier to decode orientations from a portion of the training set, and tested the classifier with the remaining data. We chose the Rank-1 General Linear Model with a 3-basis-functions HRF model, based on the fact that it yielded the highest testing accuracy score.

#### Decoding analysis

To decode stimulus types based on voxel-wise activity patterns, we used a Nu-support vector machine (NuSVC) classifier with a linear basis function implemented via the Python package *scikit-learn* v. 0.19.1 [69]. We used the de-convolved activity patterns from V1 voxels as inputs to the classifier, while trial-specific stimulus values (either orientation or spatial position) were provided as labels.

To decode orientation, we employed two separate classifiers, corresponding to the two different spatial positions (context dimension) at which the oriented gratings were presented during the training runs of the experiment (see Fig 2). Each classifier was trained to decode grating orientation (0°, 45°, 90°, and 135°) using only trials in which a specific spatial position was presented. However, the classifier was then tested with data collected from independent test runs at all four spatial positions. This resulted in an accuracy estimate at the training position, as well as at the other three spatial positions. For example, to train the first classifier, we gathered all trials that were presented at the top-right spatial position. After normalizing the data, the classifier was trained using leave-one-run-out cross-validation with data from the training runs. Cross-validation was used to optimize the *Nu* parameter of the classifier, to obtain the highest accuracies within the training set. A new classifier was then trained on all the training data using the chosen *Nu* parameter. This classifier was then tested with data from testing runs.

To decode spatial position, we employed four separate classifiers corresponding to the four levels of grating orientation (context dimension) that were presented during the training runs of the experiment. Each classifier was trained to decode spatial position (top-right vs bottom-left, see boxed stimuli in Fig 2) using only trials in which a specific grating orientation was presented. However, the classifier was then tested with data collected from independent test runs across all levels of grating orientation. This resulted in an accuracy estimate for the training grating orientation, as well as the other three levels of grating orientation. As in the orientation decoding procedure, we divided the data into independent training and test sets, performed normalization, and optimized the classifier’s *Nu* parameter via leave-one-run-out cross-validation. One important difference is that spatial position decoding involved a two-class classification problem, where the classifiers had to discriminate between the top-right and bottom-left spatial position of the stimulus window (the only two positions presented during training trials, see boxed stimuli in Fig 2). As the classifier was not trained to classify the bottom-right or top-left spatial positions, we dropped those trials from the testing data set in this analysis. This ensured that the model fitting and testing procedures remained consistent across both decoding analyses.

#### fMRI decoding tests

We applied three decoding tests to our data: the cross-classification test, the classification accuracy invariance test, and the decoding separability test. All tests where applied to the results of the two decoding analyses above: decoding of orientation and spatial position. In the descriptions below, the target dimension refers to the decoded stimulus values, and the context dimension refers to the stimulus values irrelevant for decoding that only changed from training to testing.

All the tests described below were implemented in Python expanded with *SciPy* v. 1.1.0 (https://www.scipy.org/) and *Statsmodels* v. 0.9.0 (https://www.statsmodels.org/). Plots were created using the *Matplotlib* library v. 2.2.2 (https://matplotlib.org/).

*Cross-classification test.* We implemented the cross-classification invariance test (e.g., [1–3]) by training a linear SVM, as described above, to classify levels of the target dimension (grating orientation or spatial position) while holding the level of the context dimension constant. For example, to decode orientation we start by training the SVM classifier to predict orientation in a given spatial position. Then, we test the accuracy of the classifier with data from independent test sets at the training position, as well as three other spatial positions (i.e., different levels of the context dimension). We tested whether each of these accuracies was above the chance level of 25% correct using a binomial test, and corrected the resulting *p*-values for multiple comparisons using the Holm-Sidak method.

*Classification accuracy invariance test.* This test used the same estimates of classification accuracy described for the cross-classification test, but uses them to check whether there was a significant drop in performance from the training to the testing context values. We first performed an omnibus Chi-Square test of the null hypothesis that accuracy does not depend on level of the context dimension. In addition, we tested accuracy at each testing context value against the training context value using a pairwise *z* test for proportions, and corrected the resulting *p*-values for multiple comparisons using the Holm-Sidak method.

*Decoding separability test.* Decoding separability is defined as the case where the decoding distribution of a stimulus does not change across different levels of the context dimension. The distance between the two distributions was measured through the *L*1 norm:

$$\begin{array}{c} L1=\int |p_{1}(z)-p_{2}(z)|dz, \end{array}$$ (2)

where *p*_1_ and *p*_2_ represent the distributions of decoded values at levels 1 and 2 of the context dimension, respectively.

For each combination of values of the relevant and context dimensions, we obtained decision variables from the trained SVM linear classifier. These decision variables were used to estimate the decoding distribution using kernel density estimates (KDEs). A gaussian kernel and automatic bandwidth determination were used as implemented in the *SciPy* function *gaussian_kde*. Let ${\hat{p}}_{ij}(z)$ represent the KDE for a stimulus with value *i* on the target dimension and value *j* on the context dimension, evaluated at point *z*. Each ${\hat{p}}_{ij}(z)$ was evaluated at values of *z* going from -3 to 6, in 0.01 steps, indexed by *l*, which were confirmed to cover the range of observed decision variable values. Then an estimate of the summed *L*1 distances indicating deviations from decoding separability was computed from all four KDEs obtained, according to the following equation:

$$\begin{array}{c} L1_{j}^{G}=\sum_{i}\sum_{l}|{\hat{p}}_{i1}(z_{l})-{\hat{p}}_{ij}(z_{l})|. \end{array}$$ (3)

where *j* = 1 is the training level of the context dimension. The $L1_{j}^{G}$ (with *G* standing for *global*) simply takes an estimate of the *L*1 distance (obtained by discretizing the continuous decision variable *z*) defined in Eq 2 for each value of the relevant dimension, and then sums them together. We computed $L1_{j}^{G}$ separately for each value of the context dimension, or *j* ≠ 1.

We used a permutation test to test whether each $L1_{j}^{G}$ statistic was significantly larger than expected by chance. In this test, the level of the context dimension *j* was randomly re-assigned to all data points, KDEs were estimated, and the $L1_{j}^{G}$ was computed according to Eq 3. This process was repeated 5,000 times, to obtain an empirical distribution for the statistic, from which accurate *p*-values were computed using the procedure proposed by [70]. The resulting *p*-values were corrected for multiple comparisons using the Holm-Sidak method.

### Simulations

The simulations described below were implemented in Python 2.6 extended with *Numpy* v. 1.16.2 (https://numpy.org/). The decoding analysis of simulated data was performed exactly as described for fMRI data in the sections *Decoding analysis* and fMRI decoding tests above, with the exception that the *Nu* parameter of the SVM was set to the default value of 0.5 rather than optimized based on cross-validation.

#### Model

In our simulations, we used a standard population encoding model and a linear measurement model. Both are common choices in the computational neuroimaging literature (for a review, see [43]), both in recent simulation work (e.g., [22, 47, 71]), as well as in model-based data analysis (e.g., [19, 21, 38, 72]). We assumed a circular dimension with values ranging from -90 to 90, as is the case of grating orientation, but our conclusions apply to non-circular dimensions as well.

**Encoding model.** We used standard encoding models to represent the activity patterns of populations of neurons within a given voxel. Our encoding model was composed of several independent channels, representing any number of neurons that have similar stimulus preferences. Each channel is highly tuned to a specific value along the target stimulus dimension, such that the channel’s response becomes attenuated as we move away from the preferred value. The tuning function of a single channel is represented by a Gaussian function:

$$\begin{array}{c} f_{c}\left(s\right)=r_{c}^{max}exp\left(-\frac{1}{2}{\left(\frac{s-s_{c}}{\omega_{c}}\right)}^{2}\right), \end{array}$$ (4)

where $r_{c}^{max}$ represents the maximum neural activity for channel *c*, the mean *s*_*c*_ represents the channel’s preferred stimulus, and the standard deviation *ω*_*c*_ represents the width of the tuning function. The height of the tuning functions at any value along the stimulus dimension (i.e., *f*_*c*_(*s*)) represents the average response of the channels to that particular stimuli.

We assume that the response of each channel *r*_*c*_ is a random variable with Poisson distribution:

$$\begin{array}{c} P(r_{c}|s)=\frac{f_{c}{\left(s\right)}^{r_{c}}e^{-f_{c}(s)}}{r_{c}!}. \end{array}$$ (5)

The full encoding model was composed of ten channels with activity described by Eqs 4 and 5. Unless indicated otherwise below, we used a homogeneous population model, in which the parameters *s*_*c*_ were evenly distributed across all possible values of the dimension (i.e., from -90 to 90 degrees), and other parameters were fixed to the same values for all channels: $r_{c}^{max}=10$, *ω*_*c*_ = 15.

**Fig 12:**
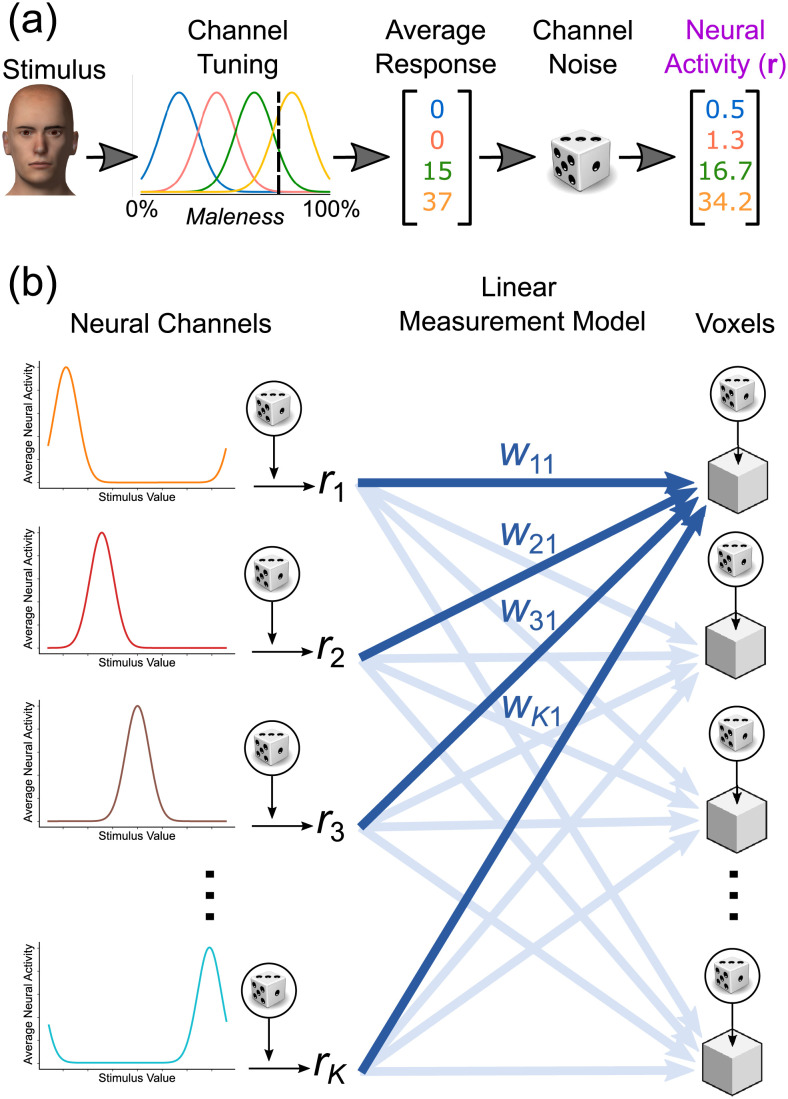
Model used in our simulations. A: Population encoding model, consisting of a set of channels that are tuned to specific stimulus values along a given dimension (e.g., maleness). When a stimulus with a particular value on the maleness dimension is presented, the channels respond according to their stimulus preferences. The channel responses are then perturbed by random channel noise. The final output represents a vector of noisy firing rates in response to a particular stimulus. B: Linear measurement model. The measurement model provides a link between neural encoding channels and voxel-wise activity measures. Activity in each voxel (represented by cubes) is a linear combination of neural channel responses. This figure includes public domain clipart and all other parts are original: https://creazilla.com/nodes/29498-white-dice-with-black-spots-clipart.

Fig 12A shows an example of the encoding process. When a face with a value of 75% maleness is presented to the model, the channel encoding distribution produces a vector of responses. Each element in this vector corresponds to the response of a particular channel. The channels with the strongest preference for the value 75% show the highest response in this vector. Since the response of neural populations are known to be noisy, channel noise is added to each element of the response vector. The final output is a noisy vector of channel responses that change slightly for repeated presentations of the same stimulus.

**Measurement model** Because neuroimaging studies produce only indirect measures of neural activity, a measurement model is required to link the neural responses of the encoding model with voxel-wise activity values. The measurement model is described by the following equation:

$$\begin{array}{c} a=\text{rW}+\epsilon , \end{array}$$ (6)

where **a** is a row vector of voxel activity values, **r** is a row vector of neural responses sampled from the encoding model (i.e., from Eq 5), **W** is a weight matrix were each column **w**_*k*_ represents the linear measurement model for a different voxel *k*, and **ϵ** is a random normal row vector with mean **0** and covariance matrix with *σ* in the diagonal and zeros elsewhere. The value of *σ* was varied in Simulation 1 and was fixed to 5 in Simulation 2 (see below).

Eq 6 indicates that the activity in each voxel is a linear combination of neural channel responses, plus some random measurement noise. As shown in Fig 12B, the model for each voxel was composed of a finite number of encoding channels that independently contributed to the aggregate signal of the voxel according to a set of weights. The values of the weights were randomly and uniformly sampled from 0 to 1, and then normalized by column, so that weights in **w**_*k*_ would add up to one. This way, the weights can be interpreted as the relative contribution of each channel to a voxel’s activity.

We simulated a total of 100 voxels. In each simulated trial, the encoding model was presented with a given stimulus and produced a random vector of neural responses **r** as explained in the previous section, which were then used as input to the measurement model to obtain a random vector of voxel activities **a**.

#### Simulation 1: False positive invariance resulting from features of the measurement model

The model underlying this simulation was created so that the encoding of the target dimension (e.g., orientation) was completely different across two levels of the context dimension (e.g., spatial position). That is, two separate encoding models were created for the two levels of the context dimension. The first context model consisted of a homogeneous population code. The second context model was composed of channels whose tuning parameters were completely randomized. For each channel, the position parameter *s*_*c*_ was randomly sampled from a uniform distribution covering all values in the dimension, $r_{c}^{max}$ was similarly sampled from values between 5 and 20, and *ω*_*c*_ from values between 5 and 25. The randomized second context model was extremely unlikely to share any properties with the first context model (compare the top and bottom encoding models in Fig 7B).

The measurement weights of the first context model, **W**_1_, were randomly sampled. On the other hand, the measurement weights for the second context model, **W**_2_, were chosen so that the activity patterns generated by any stimulus presented to this second level model would be as similar as possible as those presented to the first level model. To do this, we presented the context 1 model with the preferred stimulus of each channel *s*_*c*_ 20 times, and each time sampled data from 100 voxels. We then presented the context 2 model with the same stimuli a single time, and recorded a vector of average responses from the encoding model using Eq 4 (i.e., neural channel responses without any noise). Finally, for each voxel, the vectors of weights in **W**_2_ were obtained via Lasso regression, where voxel-wise activity patterns produced by the first context model were used as outputs to be predicted from the average neural activities obtained from the second context model. Using Lasso regression, as implemented in *sklearn*, allowed us to constrain the weights to be positive. The regularization parameter of the regression model was not optimized, but fixed to a value of 0.01.

As shown in Fig 7B, each simulation started by creating such a model (step 1), and continued by sampling data from it (step 2). To get that data, we presented the model with four stimuli, with values of -45°, 0°, 45°, and 90°, and sampled voxel activity patterns from it. Each stimulus presentation was repeated 20 times. We sampled data this way both from the first and second level models constructed as indicated above. Data was sampled twice from the first level model, to obtain training and testing data sets, and only once from the second level model, to obtain a testing data set only. We then performed a cross-classification test on the resulting data (steps 3 and 4 in Fig 7B), following the same procedures as with the experimental data explained above, with the exception that the *Nu* parameter of the SVM was fixed to the default value of 0.5. Each simulation was repeated 200 times. The results presented represent average statistics across all simulations, obtained from the testing data sets.

Finally, we repeated the group of simulations a total of 20 times, each time with a different value for the level of voxel measurement noise *σ*, going from 1 to 20.

#### Simulation 2: False positive invariance resulting from similarly tuned neural subpopulations across contexts

We created a model in which a target dimension is encoded in a completely context-specific manner, with one subpopulation of neurons responding in context 1, and a different subpopulation of neurons responding in context 2. The weights **w**_*k*_ for context 1 were randomly generated, as explained above. To create the measurement model for context 2, we first obtained a vector **e** of random values sampled from a normal distribution with mean zero and standard deviation equal to *σ*_**e**_. This random vector was added to **w**_*k*_ (step 2), and then the values were made positive through rectification and normalized to add up to one (step 3).

Once the model was generated, the simulation was carried out following the same additional steps as in Simulation 1, numbered 2 to 4 in Fig 7B. The only difference was that *σ* = 5 in the measurement model, whereas the value of *σ*_**e**_ was varied from 0 to 0.5. At the highest values of *σ*_**e**_, the standard deviation of the changes in weights in the measurement model was 500% the average value of those weights (0.1).

#### Simulation 3: On the difficulty to obtain a valid continuous measure of invariance/specificity

In Eq 2, we have used the *L*1 distance as a measure of context-sensitivity and as the basis for the decoding separability test:

$$\begin{array}{c} L1=\int |p_{1}(x)-p_{2}(x)|dx, \end{array}$$ (7)

where *p*_1_ and *p*_2_ represent the probabilistic representation of a stimulus at levels 1 and 2 of the context dimension, respectively. In the current context, the random variable *x* represents a variable or vector encoding the presented stimulus, which can be a vector of firing rates at the level of the encoding model, a vector of measurements at the level of indirect activity values (e.g., a voxel activity pattern), and a decision variable at the level of decoding.

The *L*1 distance can be interpreted as the area in blue in Fig 10. Martinez-Camblor et al. [73] proposed the complementary distance AC between two distributions:

$$\begin{array}{c} AC=\int min(p_{1}\left(x\right),p_{2}\left(x\right))dx, \end{array}$$ (8)

which is the area in red in Fig 10A. One can think of these two indexes as complementary.

We use both AC and *L*1 to define an *invariance coefficient* *ι*:

$$\begin{array}{c} \iota =\frac{AC}{L1+AC} \end{array}$$ (9)

We can compute *ι* for the encoding distributions, *ι*_*e*_, for the distributions at the level of measurement channels, *ι*_*m*_, and for the decoding distributions, *ι*_*d*_. An issue in the computation of these indexes is that some of them require integration over high-dimensional joint densities. We can estimate such integrals through MonteCarlo methods, in which an estimate of the following definite integral:

$$\begin{array}{c} \int_{a}^{b}p\left(x\right)dx \end{array}$$

is obtained by drawing *N* random samples uniformly within the {*a*, *b*} segment and calculating:

$$\begin{array}{c} \left(b-a)\frac{1}{N}\sum_{l=0}^{N}p(X_{l}\right) \end{array}$$

For an appropriate choice of *a* and *b* (i.e., such that values of *p*_1_ (*x*) and *p*_2_ (*x*) outside the interval are close to zero), the invariance coefficient *ι* is approximately:

$$\begin{array}{ccc} \iota & \approx & \frac{\int_{a}^{b}min(p_{1}\left(x\right),p_{2}\left(x\right))d}{\int_{a}^{b}|p_{1}\left(x\right),p_{2}\left(x\right)|dx+\int_{a}^{b}min(p_{1}\left(x\right),p_{2}\left(x\right))dx} \end{array}$$

we can get an estimate $\hat{\iota }$ by using the MonteCarlo estimates of AC and *L*1:

$$\begin{array}{ccc} \hat{\iota } & = & \frac{\left(b-a)\frac{1}{N}\sum_{l=0}^{N}min(p_{1}\left(X_{l}\right),p_{2}\left(X_{l}\right)\right)}{\left(b-a)\frac{1}{N}\sum_{l=0}^{N}|p_{1}\left(X_{l}\right)-p_{2}\left(X_{l}\right)|+(b-a)\frac{1}{N}\sum_{l=0}^{N}min(p_{1}\left(X_{l}\right),p_{2}\left(X_{l}\right)\right)}\\ \hat{\iota } & = & \frac{\sum_{l=0}^{N}min(p_{1}\left(X_{l}\right),p_{2}\left(X_{l}\right))}{\sum_{l=0}^{N}|p_{1}\left(X_{l}\right)-p_{2}\left(X_{l}\right)|+min(p_{1}\left(X_{l}\right),p_{2}\left(X_{l}\right))}\\ \hat{\iota } & = & \frac{\sum_{l=0}^{N}min(p_{1}\left(X_{l}\right),p_{2}\left(X_{l}\right))}{\sum_{l=0}^{N}max(p_{1}\left(X_{l}\right),p_{2}\left(X_{l}\right))} \end{array}$$ (10)

In the multidimensional case, the samples become vectors **x**_*l*_, and *p*_1_ (**x**_*l*_) and *p*_2_ (**x**_*l*_) are joint probability distributions, but Eq 10 still applies.

Because we can compute an invariance index for both encoding distributions ${\hat{\iota }}_{e}$ and decoding distributions ${\hat{\iota }}_{d}$, it is possible to evaluate the validity of ${\hat{\iota }}_{d}$ as a measure of invariance in the underlying representation ${\hat{\iota }}_{e}$, defined as the correlation between both indexes across a number of different encoding and measurement models.

We performed a simulation with that goal in mind. The encoding model consisted of only 5 channels evenly spaced along the target encoded dimension. This reduced the dimensionality of the underlying neural representation, which allowed us to precisely estimate ${\hat{\iota }}_{e}$ using the MonteCarlo procedure described above without a prohibitive sample size. The homogeneous standard encoding model was used in context 1, just as described for the previous simulation. To continuously vary the level of invariance in the encoding model, the model in context 2 was the same as the model in context 1, but each channel’s preferred stimulus and width were randomly shifted up or down by a value of *η* and $\frac{\eta }{3}$, respectively, were *η* represents the level of change in the encoding model with a change in context. The measurement model for both levels of context was built using the same procedure described for simulation 2, with *σ*_**e**_ fixed to 0.1 corresponding to the average weight value.

In each iteration of our simulation, we created the encoding and measurement models as just described, with a value of *η* randomly chosen between 0 and 6, a range of values that produced values of ${\hat{\iota }}_{e}$ between zero and one according to preliminary simulations. To estimate ${\hat{\iota }}_{e}$, we used the previously described MonteCarlo procedure with a sample size *N* = 200, 000. Each sample consisted of a random vector of neural activity values **r**, which was used to evaluate *p*_1_ (**r**) and *p*_2_ (**r**) using Eq 5 and assuming independent channels (i.e., *p*_1_(**r**|*s* = 0) = ∏_*c*_*p*_1_(*r*|*s* = 0)). According to preliminary simulations, the chosen sample size ensured convergence of the MonteCarlo estimate ${\hat{\iota }}_{e}$ to a stable value across many values of *η*. We also sampled 200 activity patterns from the measurement model at four values of the encoding dimension, including 0, and at each of the two contexts. We used these samples to estimate two versions of ${\hat{\iota }}_{d}$. For the first version, we focused on *stimulus decoding*. We used half of the sampled patterns to train a support vector classifier to decode the stimulus presented in context 1 (using the same procedures described for previous simulations), and presented the trained classifier with 100 test patterns obtained for a stimulus value of 0, presented both in context 1 and 2. The classifier decision variables obtained from those two test sets were used to estimate two decoding distributions, using kernel density estimation as described in section *Decoding separability test*. The two decoding distributions were then used to compute ${\hat{\iota }}_{d}$, using discretization of the density as described for $L1_{j}^{G}$ (see Eq *Decoding separability test*. and surrounding text). The second version of ${\hat{\iota }}_{d}$ was obtained by focusing on *context decoding*; that is, training a support vector classifier to decode the context in which stimulus 0 was presented. As before, the classifier was trained with half of the sampled patterns and then presented with 100 test patterns obtained for a stimulus value of 0, presented both in context 1 and 2. Finally, ${\hat{\iota }}_{d}$ was computed from decoding distributions obtained through kernel density estimation.

We repeated the previously-described procedure for 200 iterations, each time recording the values of *η*, ${\hat{\iota }}_{e}$, and the two versions of ${\hat{\iota }}_{d}$. We used the resulting values to compute Pearson correlations between *η*, ${\hat{\iota }}_{e}$, and ${\hat{\iota }}_{d}$.

## Supporting information

S1 Text Detailed results of the decoding tests applied in the empirical study. (PDF)
