# Supplementary material for: Improving the validity of neuroimaging decoding tests of invariant and configural neural representation
Source: PLoS Comput Biol. 2023 Jan 23;19(1):e1010819. doi: 10.1371/journal.pcbi.1010819 (PMC9894561; doi:10.1371/journal.pcbi.1010819)
Supplement: S1 Text — (PDF) [file pcbi.1010819.s001.pdf]

	Sub #1		Sub #2		Sub #3		Sub#4		Sub#5	
	Accuracy	P-value	Accuracy	P-value	Accuracy	P-value	Accuracy	P-value	Accuracy	P-value
Spatial Position Decoding										
Training Orientation 0-deg										
Testing Orientation 0-deg	95.59	<.001	100.00	<.001	95.19	<.001	81.04	<.001	95.19	<.001
Testing Orientation 45-deg	99.26	<.001	100.00	<.001	93.36	<.001	82.53	<.001	94.09	<.001
Testing Orientation 90-deg	97.76	<.001	100.00	<.001	95.93	<.001	81.18	<.001	93.70	<.001
Testing Orientation 135-deg	95.52	<.001	100.00	<.001	93.68	<.001	84.87	<.001	96.28	<.001
Training Orientation 45-deg										
Testing Orientation 45-deg	100.00	<.001	100.00	<.001	95.94	<.001	76.21	<.001	94.09	<.001
Testing Orientation 0-deg	96.32	<.001	100.00	<.001	94.81	<.001	75.46	<.001	96.29	<.001
Testing Orientation 90-deg	97.76	<.001	100.00	<.001	96.29	<.001	81.92	<.001	93.70	<.001
Testing Orientation 135-deg	96.27	<.001	100.00	<.001	94.05	<.001	76.38	<.001	95.91	<.001
Training Orientation 90-deg										
Testing Orientation 90-deg	98.51	<.001	100.00	<.001	94.07	<.001	80.07	<.001	94.07	<.001
Testing Orientation 45-deg	99.26	<.001	100.00	<.001	94.46	<.001	79.18	<.001	95.57	<.001
Testing Orientation 135-deg	94.78	<.001	100.00	<.001	93.68	<.001	80.44	<.001	96.28	<.001
Testing Orientation 0-deg	95.59	<.001	100.00	<.001	96.96	<.001	79.55	<.001	95.19	<.001
Training Orientation 135-deg										
Testing Orientation 135-deg	96.27	<.001	100.00	<.001	94.42	<.001	83.76	<.001	96.28	<.001
Testing Orientation 0-deg	95.59	<.001	100.00	<.001	96.29	<.001	83.27	<.001	95.56	<.001
Testing Orientation 90-deg	97.76	<.001	100.00	<.001	95.93	<.001	84.13	<.001	95.56	<.001
Testing Orientation 45-deg	99.26	<.001	100.00	<.001	95.20	<.001	80.29	<.001	94.46	<.001
Orientation Decoding										
Training Window 20-deg										
Testing Window 20-deg	24.81	.959	40.89	<.001	38.70	<.001	28.62	.265	34.07	<.001
Testing Window 80-deg	23.70	.988	16.00	.999	30.37	.008	23.79	.698	26.11	.643
Testing Window 260-deg	20.55	.999	27.11	.414	28.52	.068	30.48	.093	22.96	.970
Testing Window 200-deg	15.56	.999	18.89	.999	27.22	.127	25.65	.670	23.33	.970
Training Window 200-deg										
Testing Window 200-deg	35.19	<.001	54.44	<.001	35.74	<.001	32.71	.011	34.07	<.001
Testing Window 260-deg	27.22	.335	20.44	.999	28.52	.099	24.91	.786	25.92	.544
Testing Window 80-deg	22.22	.996	30.22	.021	26.11	.290	28.25	.326	24.81	.556
Testing Window 20-deg	18.52	.999	19.11	.999	27.41	.204	23.05	.789	27.96	.177

Table A: Detailed results of the cross-classification test. P-values have been corrected for multiple comparisons using the Holm-Sidak method.

	Sub #1		Sub #2		Sub #3		Sub#4		Sub#5	
	Statistic	P-value	Statistic	P-value	Statistic	P-value	Statistic	P-value	Statistic	P-value
Spatial Position Decoding										
Training Orientation 0-deg										
Omnibus Test	$\chi^2(3)$= 4.65	.19	-	-	$\chi^2(3)$= 2.34	.505	$\chi^2(3)$= 1.76	.623	$\chi^2(3)$= 2.22	.529
0-deg vs. 45-deg	z= -1.91	.16	-	-	z= .91	.739	z= -.45	.881	z= .56	.836
0-deg vs. 90-deg	z= .03	.98	-	-	z= -.42	.739	z= -1.18	.555	z= .75	.836
0-deg vs. 135-deg	z= -.99	.54	-	-	z= .76	.739	z= -.04	.967	z= -.63	.836
Training Orientation 45-deg										
Omnibus Test	$\chi^2(3)$= 5.30	.15	-	-	$\chi^2(3)$= 1.91	.590	$\chi^2(3)$= 4.12	.248	$\chi^2(3)$= 2.85	.416
45-deg vs. 0-deg	z= 2.26	.07	-	-	z= .62	.782	z= 20	.975	z= -1.19	.546
45-deg vs. 90-deg	z= 1.75	.08	-	-	z= -.21	.831	z= -1.63	.278	z= .19	.849
45-deg vs. 135-deg	z= 2.27	.07	-	-	z= 1.01	.677	z= -.048	.975	z= -.97	.555
Training Orientation 90-deg										
Omnibus Test	$\chi^2(3)$= 6.73	.08	-	-	$\chi^2(3)$= 2.13	.546	$\chi^2(3)$= .16	.984	$\chi^2(3)$= 1.53	.675
90-deg vs. 45-deg	z= -.59	.55	-	-	z= -.19	.976	z= .26	.992	z= -.79	.677
90-deg vs. 135-deg	z= 1.69	.25	-	-	z= .19	.976	z= -.11	.992	z= -1.19	.546
90-deg vs. 0-deg	z= 1.41	.29	-	-	z= -1.21	.539	z= .15	.992	z= -.57	.677
Training Orientation 135-deg										
Omnibus Test	$\chi^2(3)$= 4.04	.26	-	-	$\chi^2(3)$= 1.28	.734	$\chi^2(3)$= 1.74	.628	$\chi^2(3)$= 1.05	.789
135-deg vs. 0-deg	z= .28	.78	-	-	z= -1.03	.659	z= .15	.985	z= .43	.891
135-deg vs. 90-deg	z= -.72	.72	-	-	z= -.81	.659	z= -.11	.985	z= .43	.891
135-deg vs. 45-deg	z= 1.67	.26	-	-	z= -.41	.683	z= 1.05	.648	z= 1.01	.678
Orientation Decoding										
Training Window 20-deg										
Omnibus Test	$\chi^2(3)$= 14.49	.002	$\chi^2(3)$= 87.89	<.001	$\chi^2(3)$= 20.12	<.001	$\chi^2(3)$= 3.65	.302	$\chi^2(3)$= 22.11	<.001
20-deg vs. 80-deg	z= .35	.727	z= 8.28	<.001	z= 2.88	.004	z= 1.27	.493	z= 2.85	.004
20-deg vs. 260-deg	z= 1.38	.307	z= 4.36	<.001	z= 3.54	<.001	z= -.47	.684	z= 4.04	<.001
20-deg vs. 200-deg	z= 3.19	.004	z= 7.21	<.001	z= 4.01	<.001	z= .78	.684	z= 3.90	<.001
Training Window 200-deg										
Omnibus Test	$\chi^2(3)$= 30.83	<.001	$\chi^2(3)$= 168.77	<.001	$\chi^2(3)$= 14.49	.002	$\chi^2(3)$= 7.33	.062	$\chi^2(3)$= 13.65	.003
200-deg vs. 260-deg	z= 2.33	.019	z= 10.54	<.001	z= 2.54	.011	z= 1.99	.089	z= 2.92	.006
200-deg vs. 80-deg	z= 3.94	<.001	z= 7.35	<.001	z= 3.42	.002	z= 1.12	.261	z= 3.34	.003
200-deg vs. 20-deg	z= 5.23	<.001	z= 10.99	<.001	z= 2.95	.006	z= 2.49	.037	z= 2.17	.029

Table B: Detailed results of the classification invariance test. The symbol "-" indicates that the test could not be performed due to lack of variability in the results (all accuracies at the ceiling of 100%). P-values have been corrected for multiple comparisons using the Holm-Sidak method.

	Sub #1		Sub #2		Sub #3		Sub#4		Sub#5	
	$L1_j^G$ Statistic	P-value	$L1_j^G$ Statistic	P-value	$L1_j^G$ Statistic	P-value	$L1_j^G$ Statistic	P-value	$L1_j^G$ Statistic	P-value
Spatial Position Decoding										
Training Orientation 0-deg										
0-deg vs. 45-deg	43.25	.019	63.33	<.001	28.73	.054	31.56	.023	50.24	<.001
0-deg vs. 90-deg	45.15	.019	45.55	<.001	27.37	.061	28.67	.034	50.44	<.001
0-deg vs. 135-deg	43.41	.019	50.83	<.001	26.36	.061	20.09	.191	46.74	<.001
Training Orientation 45-deg										
45-deg vs. 0-deg	42.40	.037	27.38	.094	21.01	.156	20.51	.161	48.39	<.001
45-deg vs. 90-deg	36.48	.052	49.15	<.001	33.19	.010	39.07	<.001	40.08	<.001
45-deg vs. 135-deg	38.44	.052	53.43	<.001	29.71	.034	27.24	.056	37.13	.001
Training Orientation 90-deg										
90-deg vs. 45-deg	45.96	.017	22.69	.219	32.41	.014	30.42	.033	48.96	<.001
90-deg vs. 135-deg	31.63	.103	37.83	.007	52.84	<.001	30.63	.033	54.35	<.001
90-deg vs. 0-deg	39.77	.036	38.86	.008	30.16	.014	25.68	.040	47.40	<.001
Training Orientation 135-deg										
135-deg vs. 0-deg	50.46	.004	29.94	.040	26.47	.039	27.17	.082	54.94	<.001
135-deg vs. 90-deg	37.26	.053	44.37	<.001	49.39	<.001	27.32	.082	49.43	<.001
135-deg vs. 45-deg	38.60	.053	50.29	<.001	29.00	.039	18.93	.248	45.85	<.001
Orientation Decoding										
Training Window 20-deg										
20-deg vs. 80-deg	104.51	<.001	101.05	.004	65.32	.208	74.28	.713	72.73	.163
20-deg vs. 260-deg	76.81	.307	90.32	.016	90.96	.008	87.72	.504	49.88	.688
20-deg vs. 200-deg	71.37	.199	187.63	<.001	109.78	<.001	63.73	.719	86.35	.024
Training Window 200-deg										
200-deg vs. 260-deg	114.29	.006	281.98	<.001	79.34	.031	106.29	.082	78.88	.068
200-deg vs. 80-deg	102.44	.001	208.15	<.001	105.77	<.001	92.98	.125	95.57	.004
200-deg vs. 20-deg	91.25	.006	314.68	<.001	84.35	.026	106.27	.082	69.58	.120

Table C: Detailed results of the decoding separability test. P-values have been corrected for multiple comparisons using the Holm-Sidak method.
